# From chisel to inscription: affordable protocols for the digital documentation of stone carving techniques. An experimental archaeology and traceological approach applied to epigraphy

**DOI:** 10.1371/journal.pone.0327303

**Published:** 2025-07-07

**Authors:** Giulia Previti, Salma Kusumastuti, Beatrice Luci, Luca Malatesta, Cristina Lemorini

**Affiliations:** 1 Department of Sciences of Antiquity, LTFAPA Lab, Sapienza University of Rome, Italy; 2 Museum Universitas Gadjah Mada, Yogyakarta, Indonesia; 3 Department of Environmental Biology, Sapienza University of Rome, Italy; Universita degli Studi di Ferrara, ITALY

## Abstract

This study investigates technological traces in stone epigraphs to reconstruct the methods, tools, and gestures used by artisans. It aims to analyze the techniques behind these inscriptions, highlighting skills, challenges, and interactions between craftspeople and stone, as well as territorial differences. Experimental archaeology enables the creation of a reference collection of replicated inscriptions, providing a comparative framework for technological trace analysis. By integrating experimental archaeology with traceological analysis, this research introduces a novel methodology for epigraphic studies through qualitative and quantitative approaches. A key contribution is the use of micro-photogrammetry as a non-invasive, non-destructive documentation technique, particularly valuable for fieldwork. This method enables high-resolution, meso-scale recording of inscriptions, even on immobile surfaces, common in epigraphic studies. Both qualitative and quantitative analyses are applied to interpret technological traces, including fatigue and abrasive wear. These traces reveal information on the direction, depth, and angle of engraving, shedding light on artisans’ techniques and challenges. Quantitative methods refine the analysis by providing precise insights into the engraved surface topography and roughness. Moreover, slope analysis clarifies tool orientation and movement, enabling the visualization of trace profiles and validating qualitative observations.

## 1. Introduction

This paper discusses the informative potential of traces analysis to reveal the technological traits that characterize the inscriptions in the past. How a letter is crafted in the stone, the kind of movements made to engrave, the tools used, the possible errors and the possible actions to adjust it are all expressing the cultural embodiment that the artisan materializes in his/her writing. The manipulation and transformation of the environment is one of the defining features that has distinguished humankind since the dawn of humanity. In this sense, technology has played a central role in human behavioral and cultural development.

In the past century, André Leroi-Gourhan coined the term *“geste technique”* [1] to emphasize the fundamental role of human motor skills in technological processes. By documenting traditional activities and reproducing them through experimental archaeology, Leroi-Gourhan, and later other scholars [2], initiated a broad reflection on the relationship between the mind, gesture, and learning which more recently has broadened to also encompass the relationship with the material being worked [3]. Alongside the study of the *“geste technique”* through ethnoarchaeological and experimental approaches, traceology, by detecting the marks left by human gestures on materials during their manipulation and transformation, offers a means to deepen our understanding of past gestures, revealing and reconstructing the actions behind the traces.

Thus, the documentation of craftsmanship as tangible expression of the technology, in modern days and in the past, is essential to define the cultural traits of the human communities and to reveals the connections between applied techniques and cultural behaviors and norms.

The agency of the artisans and the workshops dealing with inscriptions is readable through the traces left by the instruments used to produce the inscription itself. Scrutinizing inside the grooves that compose each single letter by a magnified observation allows us to intercept the morphological features that document the type of tool used and the artisan’s gesture. Traces analysis may decipher not only the general direction followed by the artisan to produce a single letter, but as a series of factors that influence the graphic representation of the inscription, such as the inclination of the instrument, the position of the artisan in relation to the inscription, the spacing of the engravings, corrections and revisions on the same section, and any difficulties encountered in the production of the artefact, dictated, for example, by the mineralogical composition of the raw material.

Moreover, restricting the analysis to a macroscopic examination may make incorrect or, in any case, incomplete observations. As an example, the weathering of the stone surface is not always visible to the naked eye. The microscopic examination may be the only way to document the degree of preservation of the stone surface and the technological traces eventually not affected by the alteration processes.

In this paper we demonstrate that the documentation of technological traces observed and documented at the mesoscale allows to reconstruct the actions carried out to produce the inscriptions, obtaining unvaluable data to recognize the techniques applied, the skill of the artisan, the possible involvement of individuals with different expertise.

Linking the traces identified on the stone with the actions and gestures made by people helps to explore the concept of craft work and *chaîne opératoire* [4,5]. In archaeology, this term refers to the study and reconstruction of production organization [6] linked to raw material exploitation, expertise, commissions, economy [7]. Recognizing craft workshops often involve identifying recurring materials, products, activities and gestures [8]. Skill, for example, can be identified through the accurate execution of steps, the ability to easily repeat actions, and the capacity to work with raw materials using the most effective approach [9]. According to these principles, it is possible to outline the profile of a *magister* or groups of craftsmen active in specific geographic areas, highlighting phenomena of contamination or local productions.

Here, we propose, for the first time, the application of an integrated approach combining experimental archaeology and traces analysis to reconstruct the engraving techniques applied on the production of stone inscriptions.

Traces analysis allows us to decipher the signs left by the tools on the worked material and to interpret the production sequence. Experimental archaeology allows us to replicate these signs resulting in a reference collection to compare with the sample studied [10].

Technological traces are mainly invisible through macroscopic observation. Observation at the meso- or the microscale are essential for a correct application of the method. It implies the use of optical or digital microscopes to magnify the sample, usually in a laboratory setting [11]. Recently, given the need for detailed documentation in the field, we successfully applied a new non-invasive and non-destructive technique of documentation of technological traces combining the use of a portable digital microscope with micro-photogrammetry [12]. As a result, the technological traces are easily documented with the needed magnification and details also in field and on non-mobile surfaces, that is the most frequent condition of the stone inscription.

In this paper we discuss the achievement of the protocol that allows to document the engraving techniques applied to the stone inscriptions and we show the first results of its application on two medieval epigraphy from Central Italy. We demonstrate that the documentation of these features allows to reconstruct the actions carried out to produce the inscriptions, obtaining unvaluable data to recognize the techniques applied, the skill of the artisan, the possible involvement of individuals with different expertise.

In perspective, this contribution aims to provide a research method to study inscriptions. As an example, in central Italy and particularly in northern Lazio, these inscriptions should also be examined in relation to their connections with Rome and its patrons during the early and late Middle Ages, combining archaeological data from historical sources with material evidence that marks the evolution of execution techniques and writing styles.

### 1.1. State of art

In the history of studies many efforts were made by various scholars to enhance the readability of inscriptions suffering erosions that may affect the recognition of the letters and, thus, preventing to understand partially or entirely the meaning of the text. In fact, in the field of epigraphy, the graphic representation of an inscribed artefact has always been necessary, because for a complete understanding of the text it cannot be separated from its support, and traditionally the manual squeeze (or apograph) was the first method used [13]. With the introduction of photography and new graphic programs (e.g., AutoCAD, Adobe Illustrator Draw, Gimp), the manual squeeze was flanked by the digital squeeze, which has the advantage of overcoming the two major limitations of the manual squeeze (the impossibility of always being in direct contact with the inscription, either because it is in an inaccessible place or because the inscribed surface is compromised to such an extent that it could be damaged by the action of the squeeze) [14].

In their article, Seales and Chapman [15] summarize the impressive technical advances in digital analyses that nowadays are applied to the study of grooved and painted epigraphy.

Indeed, since epigraphic studies entered the world of technology, countless digital experiments have been carried out. Until now, photogrammetry (3D) and RTI (2D) primary and ultraviolet and, occasionally, infrared light has been the most common methods used in the field [14–21].

As well as being particularly communicative even for a non-expert audience, the 3D model allows not only greater intelligibility of epigraphic texts but also full understanding of the close relationship between inscription and its support, and thus between epigraphy, archaeology, and art history. On the other hand, RTI has the great advantage of highlighting, with a non-invasive technique, details not visible to the naked eye and often not visible even with photogrammetry in the case of inscriptions in which the epigraphic text is not perfectly legible due to the poor preservation of the surface of the support (as is often the case with the high leveled surface of epigraphs engraved on paving slabs) or due to the engraving technique that may not be too well readable (as in the case of graffiti technique).

Although it is totally understandable that the effort made in improving the methods of reading that allow to reconstruct and to decipher the written text, few research was addressed to reveal the engraving technology behind the inscriptions. In fact, existing studies show that the identification of the artisan’s hand has primarily focused on stylistic and macroscopic features. In contrast, our method reverses this approach by launching a traceological investigation at the mesoscopic level, gathering clues that may pinpoint individual artisans or the associated workshops.

With regard to specific writings on Western epigraphic material (Latin and medieval), especially for the Classical period, there are several references to the instruments used [13,22], whose identification is mainly based on the study of written and iconographic sources, as well as the macroscopic observation of the signs impressed on the stone, such as the type of incision (mainly with a ‘V’ section and with a ‘U’ section). The attempt to read traces at a magnified level using digital microscopy techniques is instead limited to analyzing the incision profile, measurements, depth and irregularities [11,23]. These same variables are the ones considered in the application of an innovative study on engraved runes using 3D scanning and multivariate statistical analysis to understand the carving technique, although this does not take into account the micro-traces visible inside the incision [24]. At the same time, in recent years, the use of experimental archaeology to implement knowledge of production processes in the epigraphic field has also begun to expand: in some cases, for example, experimental archaeology has been combined with the macroscopic observation of the main morphological characteristics (e.g., the size of the letters) describing the inscription [25]; in others [26], the technological traces obtained with experimental archaeology have been compared at the macroscopic level with those observed on the archaeological material, deriving information such as the tool used and the position of the engraver’s body, also making use of the author’s professional experience in the field of stone working [27], as well as comparison with iconographic sources.

Based on what has been discussed, it is evident that, at present, specifically in the case of epigraphic material, only the technological traces visible to the naked eye have been studied, including the shape of the incision of the letters, and from these the instrument used to engrave the text has been identified by comparison with the bibliographical and iconographic material, and occasionally with the help of experimental archaeology. However, the groove of each inscription has not yet been subjected to microscopic analysis in order to highlight the various technological traces that characterize the inside of the groove. Thus, there is a clear lack of a combined use of experimental archaeology and tracelogical analysis, which would allow for the interpretation of incisions and the reading of traces by merging qualitative and quantitative analysis with a higher level of detail and observation scale.

This type of study is necessary to investigate in details certain issues, such as the characterization of the artisan gestures that allow to decipher the techniques used to engrave each single part of the letter and the alteration of the surface of the support, which are fundamental for the correct reconstruction of the production cycle of the inscriptions, as well as for the possible identification of the respective production workshops, with obvious repercussions for the reconstruction of social relations and artistic influences between the different communities, both small and large.

## 2. Materials and methods

### 2.1. Archaeological materials

Regarding the selection of archaeological materials (for which only one letter from each was chosen for analysis), two inscriptions were chosen as samples, both originating from the same topographical context (the province of Viterbo, north of Rome) and lithic composition (both made of marble). The goal is to verify if, trough an experimental and traceological approach, it is possible to detect differences and/or similarities in the technological production of the epigraphs between the early and late Middle Ages (the first inscription dating to the 9th century, the second to the 13th century) within the same geographical and raw material framework. Additionally, the study aims to assess whether the depositional processes suffered by the inscriptions led to significant differences in the alteration of the inscribed surfaces and how these changes might have affected the visibility of the traces.

The first inscription was above the main eastern entrance of Leopoli-Cencelle, a papal foundation town in Italy, north of Rome, in the province of Viterbo, which has been systematically investigated by the Sapienza University of Rome since 1994. Leopoli-Cencelle was founded by Pope Leo IV in the 9th century and abandoned in its urban function in the 16th century [28,29].

The monumental inscription was commissioned by Pope Leo IV (847-855) for the founding of the city and it reflects the political circumstances that prompted him to undertake this initiative: the epigraphic text clearly shows the primary need to create a city that would protect its inhabitants from enemy threats (*Leonis q(uarti) papae* ((crux)). *Quamvis in parvo co[ns]istat condita [loco] urbs haec nulla hominum se[d bel]la nocere va[lebunt desinat hinc bellato[r atrox iam desinat hostis non hanc ut [quicumque potes]t urbem violare*). In fact, the city was founded to serve as a safer refuge for the citizens of *Centumcellae* (modern Civitavecchia, located in the province of Rome), who at the time were under attack from the Saracens along the Tyrrhenian coast [30].

The inscription was found a few years before 1900 [31] at the eastern entrance [30] and, kept at the Municipality of Civitavecchia, was significantly damaged during the bombing of the Second World War and is now preserved, fragmented and lacunose, in the storage rooms of Forte Michelangelo in Civitavecchia (Province of Rome).

The text is engraved with a triangular groove on a partially preserved marble tabula ansata (6 fragments plus two known from previous photographs. The text is inserted within a rectangular vimineous ribbon frame that runs along the limits of the tabula and the writing is an epigraphic capital letter with a strongly verticalized letter module (7.5 cm height x 3 cm length).

The text is evenly distributed on the support and reflects particular care in the layout, including the insertion of two monograms at the two handles of the tabula.

At a macroscopic inspection, the inscription presents a good state of preservation of the surface: only a slight smoothing can be observed.

**Fig 1:**
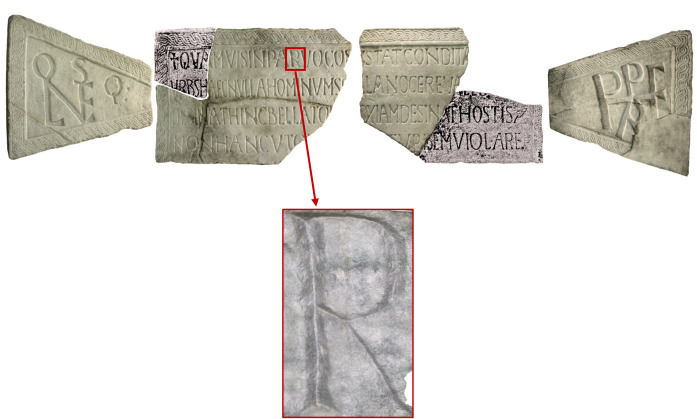
A monumental inscription commissioned by Pope Leo IV (847-855) for the founding of the city of Leopoli (Cencelle). Highlighted is the letter R of the word ‘parvo’, selected for traceological analysis.

The letter R of the word ‘*parvo*’ (first line of the text) was selected for the analyses (Fig 1).

The second inscription, on the other hand, comes from the city of Viterbo, located north of Rome as Leopoli-Cencelle. The text is engraved with a triangular groove on a block of marble consisting of a base, where the epigraph is inscribed, and a sphinx sculpture; the epigraphic text reveals the signature of the sculptor who created the sphinx in 1286: ((crux)) *Hoc opus fecit f(rate)r Pascalis Roman(us) Ord(inis) P(re)d(icatorum) a(nno) D(omini) MCCLXXXVI*. In the 17th century the sculpture was placed in front of the left column of the funeral monument of Pietro di Vico [32], one of the leading figures of the Viterbo political scene, in the cathedral of S. Lorenzo, also in Viterbo. Currently, it is part of the exhibition in the Civic Museum of Viterbo. The text is positioned along the entire surface of the support, therefore it is rectangular in shape; the letters are of a constant module as far as height is concerned (about 4 cm), while for the width one notices a narrowing in the second part of the text due to the lack of space on the support (from 1.3 cm to 0.9 cm). Having likely miscalculated the layout space the artisan was obliged not only to restrict the letters but also to reduce the spacing between individual words and increase the abbreviations of the words. With regard to the letters, it should be noted that almost all of them are decorated with elongated apicatures that continue beyond the line and always end with a curl. The script is a ‘Gothic capital lettering’ typical of the Viterbo area, generally characterized by the following elements: ‘A’ with the crowning stroke shifted to the left; capital ‘D’; round ‘E’, ‘M’ and ‘U’; curly ‘G’; lowercase ‘H’ and ‘N’; ‘T’ with the ‘a falce’ form, i.e., with the vertical stroke curved and not straight [33,34].

At the macroscopic inspection, the inscription is in a good state of preservation even if a light sheen perceivable on the surface suggests a possible intervention of restauration by polishing, with an abrasive white substance still visible in some areas of the engraving. It can be hypothesised that the procedure is related to an attempt to restore the artefact, which may have been damaged during the Second World War, in line with what happened to numerous heritage sites in the area around Viterbo.

**Fig 2:**
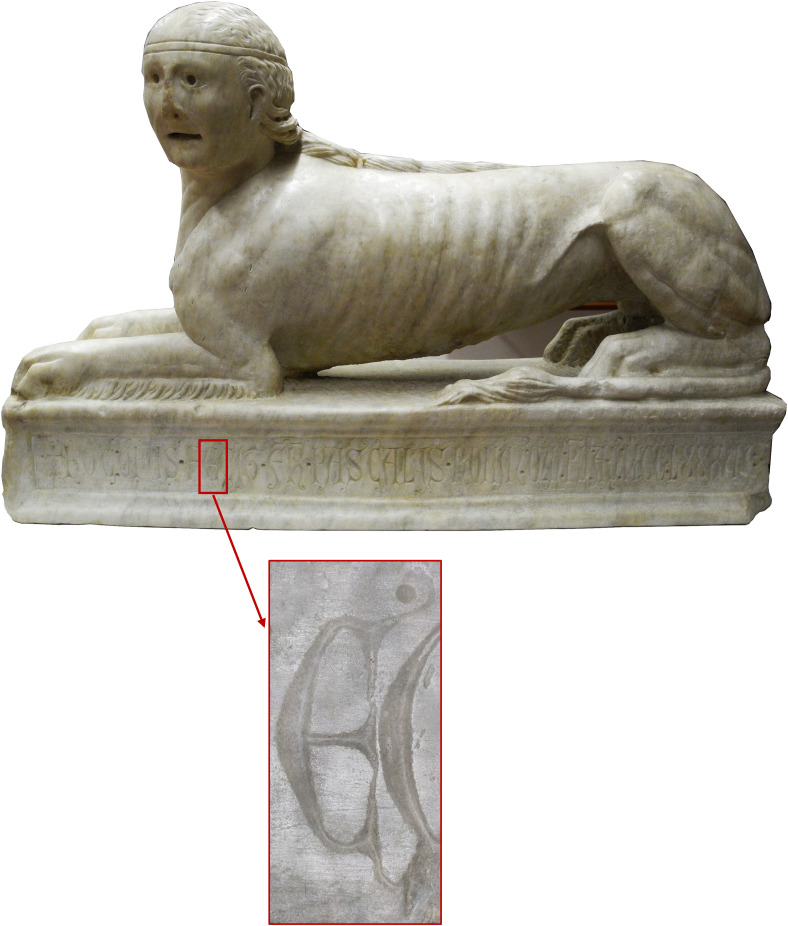
A 13th-century inscription, housed at the Civic Museum of Viterbo. The text is engraved on a marble block that forms the base of a sphinx sculpture. Highlighted is the letter E of the word ‘fecit’, selected for traceological analysis.

The letter E of the word ‘*fecit’* was selected for the analyses (Fig 2).

### 2.2. Methods

#### Experimental archaeology.

**Fig 3:**
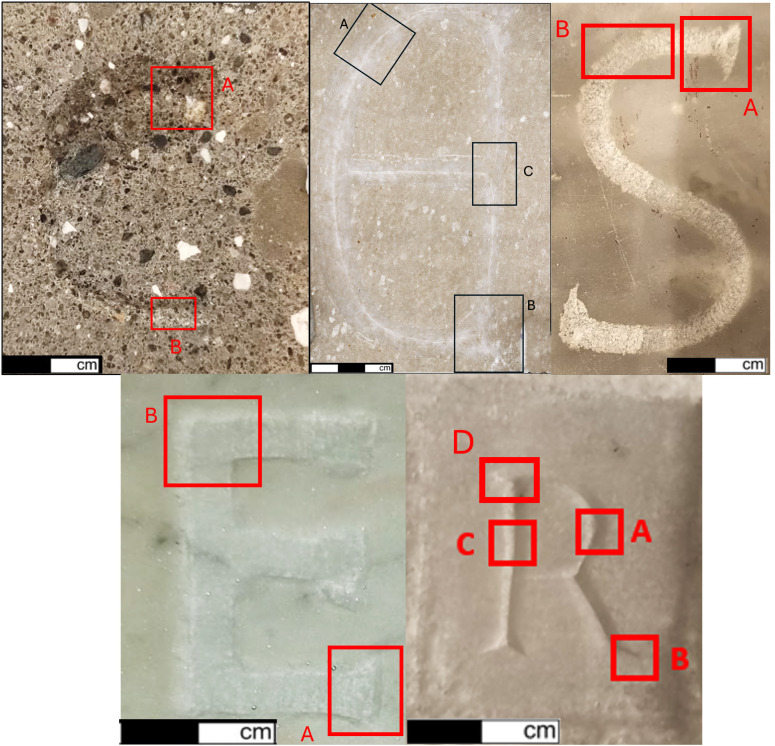
Experimental reference collection: letters C, E (Gothic), S, E (Capital), and R. The letters were engraved on square slabs of various types of marble, except for the letter C, which was made using volcanic tuff, traditionally known in Italy as peperino. For each letter, the parts selected for traceological analysis are highlighted, always corresponding to a portion of the body and the final part of the letter.

In this research, experimental archeology was applied to generate a reference collection of letters engraved in stone replicating the main epigraphic styles documented in Roman and Medieval ages in Central Italy (V-shape groove, U-shape groove and square groove). The letters C, E (Capital), E (Gothic), R, S, presenting the morphological features most representative of each style, were replicated (Fig 3). The letters were all engraved in single square slabs of marble apart from one case where the volcanic tuff, traditionally known as peperino in Italy, was used. Marble is the most common raw material employed for stone inscriptions, especially during Roman age. *Peperino* is a tuff typical from the Central Italy documented as slab for inscriptions in Roman age and, especially, in Medieval age. *Peperino* has a highly coarser texture, compared to marble, due to its inhomogeneous structure reach of large inclusions.

An artisan expert in calligraphic replicas, both in stone and in parchment, carried out the engraving using metal tools that are exact copies of the Roman stone-carving toolkit, based on the iconographic and archaeological sources, such as those found in Pompeii. These tools remained similar in later periods and can also be considered valid for the Middle Ages. [35–37]

**Fig 4:**
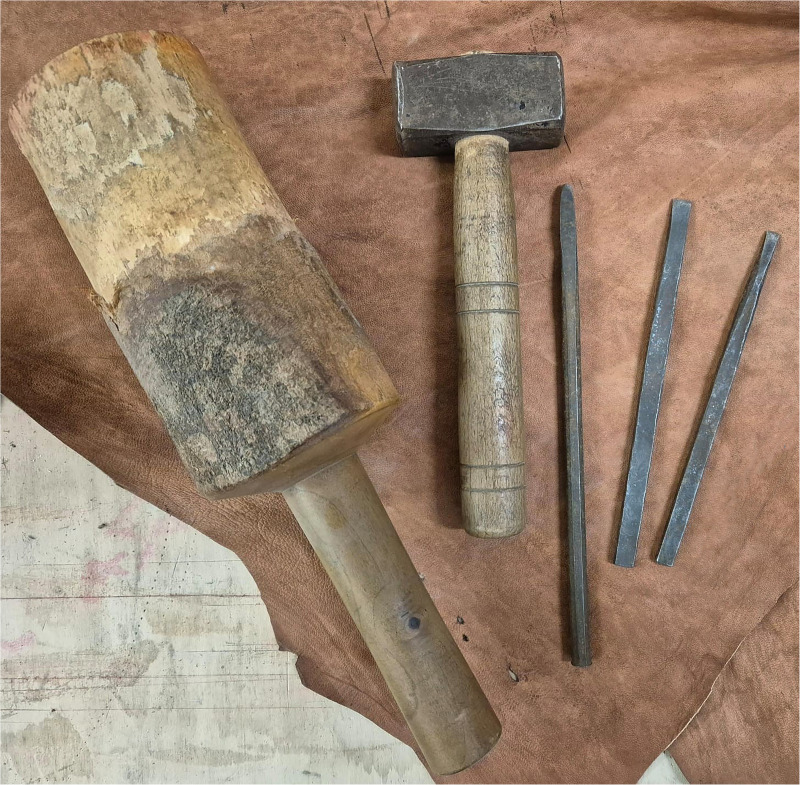
Experimental replicas of iron tools. The experimental activity was carried out using iron tools that exactly replicate the traditional equipment for working stone, as described in Roman and Medieval sources. In the photo, from left to right, two types of hammers are visible – one wooden and one metal – a round chisel, traditionally known as an *unghietto*, and two flat-tipped chisels of different sizes.

The replicas of the tools used include two types of hammers – one made of wood and one of metal -two flat-tipped chisels of different sizes, and a rounded chisel, traditionally known as *unghietto* (Fig 4). Each experimental session was documented with video and pictures.

The experiments were aimed at documenting the technological traces originated during the production of each single letter and to relate them to the tools used to their inclination during work, to the direction of the engraving activity, all parameters essential to infer the techniques put in place. These traces are generally not visible at the macroscopic observation except for those inscriptions composed by very large letters and very large incisions allowing to observe by naked eyes or with the auxilium of a camera with macro lens some of the more visible traces left on stone surface and on the incision itself. In many other cases the incisions barely reach 1 cm of width and some mm of depth implying the use of equipment allowing to magnify the observed surface at the mesoscale [12].

#### Qualitative analysis.

To analyse the reference collection of letters, we applied the observation at the mesoscale using three approaches: 1- stereomicroscope Nikon SMZ with oculars 10X and objective 0,5X with a magnification zoom ranging from 0,75X to 75X in reflected oblique light with a fiber optics system (Photonic F1-set, consisting of the F1 LED cold light source at intensity 2 and a fiber-optic light guide featuring a double-arm gooseneck); 2 –same stereomicroscope in transmitted light; 3 – micro-photogrammetry using a portable digital microscope Dino-Lite Edge AM7915MZTL with a magnification range from 10× to 140x and equipped with a long working distance (LWD).

The three approaches were carried out to compare the readability of the technological traces using the techniques developed in laboratory conditions (stereomicroscope) and in field conditions (micro-photogrammetry). In a previous paper [12], we already demonstrated that micro-photogrammetry coupled with portable digital microscope is the optimal non-invasive and non-destructive technique to document technological traces at the mesoscale in field condition and, especially, to document non-mobile samples and surfaces. Here, we wanted to go deeper in verifying the readability of this technique to detect the technological traces of stone inscriptions by comparison with the standard modality used in laboratory. In the laboratory, we observed directly the surface of the replicas of the engraved letters with the stereomicroscope in reflected light mode. Furthermore, we observed resin casts (Araldite LY 554 and Hardener HY 956, in an 80%/20% ratio) of informative portions (end, body) of the incisions composing the letters in transmitted light. This technique allows to obtain an increased view of the details of the technological traces, better than the direct observation of the sample. The only problem related to the use of resin is the formation of bubbles that can disturb the observation of the surface. However, the experience gained during this study confirmed that the presence of few scattered bubbles does not prevent documentation. Nevertheless, despite the very good results obtained applying mould and cast techniques on replicas, moulding technique is risky if applied on the original stone surfaces since it can originate discolored spots in the areas where the silicone was applied.

For this reason, to analyze sampled letters from the two medieval epigraphy used as a testcase, we operated exclusively with micro-photogrammetry coupled with the digital microscope Dinolite.

The replicas were documented at the LTFAPA laboratory of Sapienza University of Rome, and the archaeological inscriptions were documented at two locations: one at the Civic Museum of Viterbo and the other at the Fortress Michelangelo of Civitavecchia, where materials from the Cencelle (VT) excavation are stored.

The replicas were washed with demineralized water using a pressure spray to clean up the particles of stone accumulated in the incisions during the stone working. After drying, the washing procedure was refined using compressed air spray.

Due to the impossibility to use water in the exhibition area, the epigraph kept in the museum was cleaned exclusively with compressed air spray. The one stored in an archaeological warehouse, not on public display, was cleaned with both demineralized water and compressed air after it dried.

The replicas, given the convenient working position in the lab, were documented using the basic stand of the portable digital microscope, which has an adjustable horizontal arm allowing 360-degree rotation to tilt the microscope in various directions. In this case, we tilted it by 1 cm from a starting position of 0 degrees. The object to be documented was placed vertically on an x-y stand table, which, thanks to knobs, enables precise, controlled, and repeatable movement along the x and y axes. The distance between the documented object and the microscope was 6 cm, which proved to be the optimal measurement for traces documentation. For all marble replicas, the microscope’s light setting used a combination of maximum polarized light and LED settings. This condition enhances the crystals and stone components, which become overlapping points for photogrammetric documentation. This setting was chosen only for white and light pink marble, as light surfaces are very reflective; for peperino, however, the light mode uses only two lamps on the microscope, creating a grazing light from two different angles.

The documentation of archaeological inscriptions was carried out in a depot and a museum, in what can be described as field conditions. The lighting in these spaces was not an issue, thanks to a multi-purpose cone (an accessory available with the digital microscope, 12 cm long) used to shield the portable digital microscope from external light. For both archaeological samples and replicas, a 6 cm cone was used, redesigned based on the 12 cm model and created with a 3D printer. This shorter cone allowed for much more precise and effective detail capture in the 3D model. Although this shorter distance increased the time needed for each scan compared to the 12 cm distance [12], the resulting quality was far superior. Here, too, the microscope settings combined maximum polarization with LED lighting, as the white surfaces being analyzed produced significant reflections.

The photos were taken using DinoCapture 2.0 software, which is free for Dino-Lite users and required for capturing and exporting microscope images. They were then processed with Agisoft Metashape Professional software (version 1.8.5 build 14752, 64-bit) on a laptop equipped with a 13th Gen Intel(R) Core (TM) i7-13700HX processor and 32.0 GB of RAM. For both processes, refer to the workflow followed in Previti et al. (2024). The result is a 3D model that can be viewed in both mesh and texture modes. For an accurate analysis of the traces on the three-dimensional model, the mesh has proven to be much more suitable. Without the image map projected onto the polygons, it allows for a direct view of the worked surface of the stone. The software allows for zooming into the model, starting from the measured view (Reset View). For an accurate evaluation of the traces, it is possible to zoom in until the mesh remains dense and individual polygons are not visible. The model can be navigated until the overall surface view is lost and the underlying mesh structure becomes apparent. The maximum zoom level varies from model to model, depending on the density of the point cloud and the processing work performed and from the level of detail of the photogrammetric documentation.

An important choice was determining the area to document: for the replicas, the entire letter was captured to gather as much information as possible on the production phases. For the epigraphs, for sake of time, only one letter from the entire inscription was selected and documented by micro-photogrammetry.

This selection was carried out by means of a macro-photogrammetric survey of the entire epigraph, combined with a visual examination to identify s key areas of interest avoiding the less preserved letters.

#### Quantitative analysis.

For quantitative analysis of the microtopography, 3D objects created in AgiSoft MetaShape have been exported as Digital Elevation Model (DEM) raster grids at 0.015 mm spatial resolution (side length of the single square pixel) and assigned a custom metric coordinate reference system. DEMs are raster images in which each pixel represents a portion of the object surface (in this case, 0.015 mm*0.015 mm = 0.000225 mm2) and contains a numeric value representing the elevation of that portion with respect to a “zero” level (i.e., sea level in case of geographic data, base plane in our specific case).

Slope and roughness of the surface have been computed applying neighbourhood analysis techniques based on a moving window of 9x9 pixels. With this approach, each pixel value is compared with the values of the 8 neighboring pixels to compute slope and roughness values. Slope is the angle (expressed in degrees) of inclination of the surface with respect to the horizontal, calculated as arctan(∆e/l), where ∆e is the difference in elevation values between two neighbouring pixels, and l is the length of pixel sides (spatial resolution of the DEM raster). Roughness is the largest inter-cell difference between the elevation values of a central pixel and its 8 surrounding cells. To obtain slope and roughness layers we used the “Slope” and “Roughness” algorithms in the “Raster analysis” package of the Geospatial Data Abstraction Library (GDAL), a translator library for raster and vector geospatial data formats [38].

Terrain profiles along opportunistic lines of interest have been created based on DEM values using the “Profile Tool” plugin (https://github.com/PANOimagen/profiletool) in Quantum GIS. All the spatial data processing and analyses have been conducted using Quantum GIS version 3.30 “s-Hertogenbosch” [39].

## 3. Results

### 3.1. The reference collections

Each letter was carved on the stone slab of marble (R, S, E Capital and E Gothic) and peperino (C) (Fig 3). The procedure was carried out in a standing position where the slab was placed on a wooden table; the hammer was gripped by the right hand and the chisel on the left hand. Regarding the hand gesture, the artisan tried to manage constant strikes at a specific chisel angle. However, the angle is adjustable depending on the materials and the letter part. The stability of strikes was often disturbed by the stone displacement and the physical state of the stone surface. Another cause of interruption was the constant cleaning of the deposit of debris accumulated on the groove during work.

According to the working gesture, some considerations should be mentioned for the different starting and ending points of work, the changes caused by the direction of the work, and the specific strike stress for selected points.

#### The letter R.

Generally, the letter R has three parts: the stem, arch, and leg. The artisan attempted to reproduce the Roman-style R on a white marble surface for the experiment (S1 Video; S1 Text). The process began by designing the letter with a pencil before carving it using a flat chisel and a wooden hammer. The result was a letter with a V-shaped groove formed by two diagonal walls on the letter.

**Fig 5:**
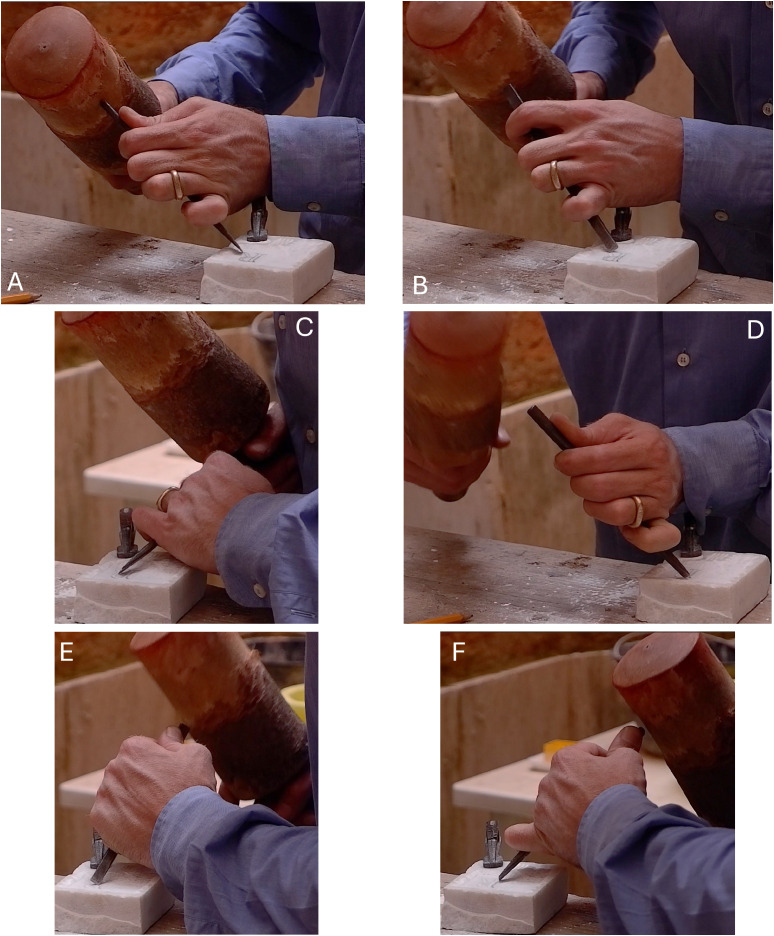
Stages of crafting the letter R by the artisan. A: shows the starting point from the leg, with the chisel placed at a 45° angle. B: illustrates the rotation movement performed when the chisel reaches the curved part of the leg. C: represents the execution of the arc, where the chisel’s corner tip is mainly used at nearly 45°. D: shows how the chisel gradually shifts and settles into a wider angle at the end. E and F: depict the creation of the stem, starting from the inner part and then shaping the outer part.

The leg is the first part to be formed. It has a slightly curved feature on the lower part. As the starting point, the artisan established the chisel corner tip on the outermost side of the leg. The chisel was placed at 45° and beaten with a wooden hammer (Fig 5A). A series of uninterrupted strikes commenced and constructed the outer wall of the letter. The rotation movement was noticed when the chisel arrived at the curved part of the leg (Fig 5B). He made a similar gesture to incise the inner wall. Besides a similar gesture, equal repetitions were applied to complete the construction of both walls.

The arch was carved from the lower to the upper part. The chisel corner tip was predominantly used at nearly 45° (Fig 5C). As it reached the upper part of the arch, the chisel gradually shifted and established into a wider angle at the end (Fig 5D). The artisan took more strikes for the end of the upper part (serif). For the wall forming, the outer wall was carved more extensively than the inner wall.

The stem consists of a serif on the lower part, formed by opposite diagonal lines corresponding to the outer and inner walls. The stem was created with an upward motion, starting from the innermost part of the serif to shape the inner wall (Fig 5E). Then, a slight rotation and repeated strikes upward shaped the outer wall (Fig 5F). The strength of the strikes was recognized as a way to develop the serif, especially for the inner wall.

#### The letter S.

**Fig 6:**
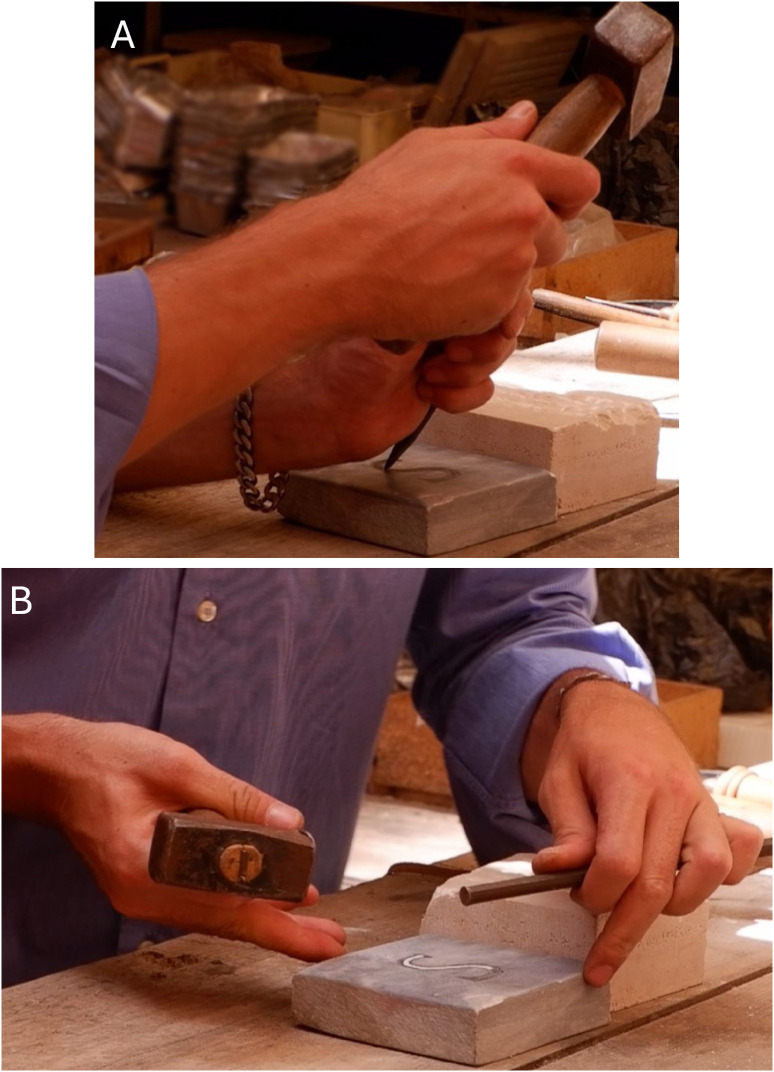
Stages of crafting the letter S by the artisan. The figures show the rounded tip chisel and squared metal head hammer during the carving process, performed in various working directions. It is possible to see that the chisel is kept at a 45° angle throughout the entire process.

The artisan designed the letter S based on one of the medieval calligraphic styles that uses U-shaped carving instead of the more common V-shaped carving. A rounded tip chisel and squared metal head hammer were employed to accomplish the letter on a piece of gray marble (S2 Video; S2 Text). During the carving process, the artisan rotated the piece between 180° and 90°. As a result, the letter has been completed in various working directions. For example, the observed part, which is perceived as the lower half of the arch in the initial position, attempted at least four types of motion: horizontally towards the edge, horizontally toward the arch, upside down, and bottom-up. Furthermore, specific moves intensified the edge part to be deeper and have a convex outline at both ends. The chisel position was maintained at 45° with the steady state of strikes, despite disruption amid the arch shaping (Fig 6A–B).

#### The letter C.

The letter C was engraved on the piece of tuff (*peperino*). Unsorted grains are visible, as represented by the surface roughness. The artisan utilized a flat chisel and squared metal head hammer (S3 Video; S3 Text). Several turns in 90° were enforced to follow the layout of the half-circle-shaped letter C. A series of strikes displaced the piece, so the carving process was occasionally interrupted to fix the position and clean the debris. In this case, having a small slab not fixed to the work surface, combined with stronger strikes required due to the type of stone, made the carving process more difficult.

**Fig 7:**
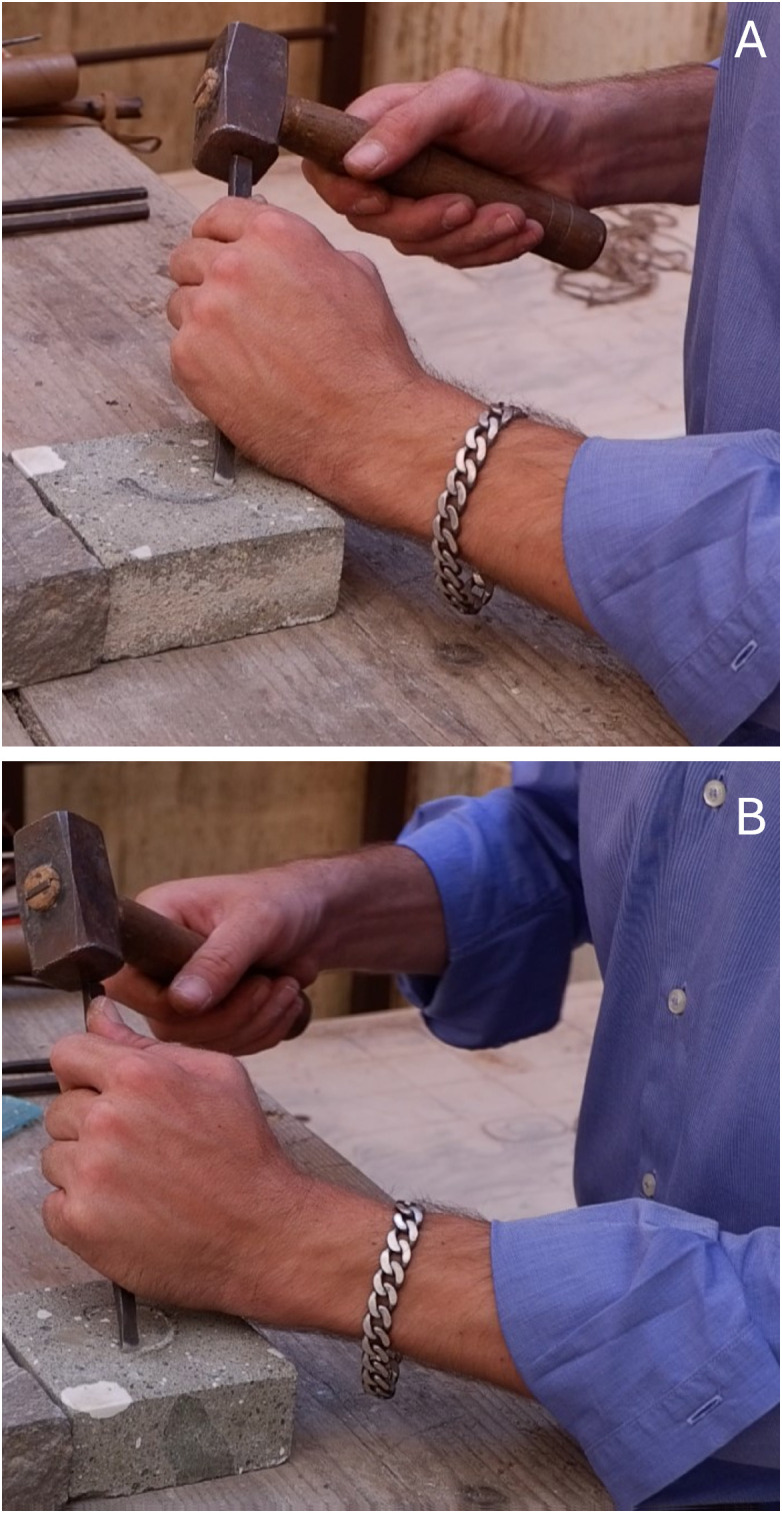
Stages of crafting the letter C by the artisan. A: shows the engraving path from the lower half-edge to the middle arch, with the chisel held at a 45° angle; B: shows a slight rotation of the chisel to carve the upper and lower edges.

The letter C could be divided into two parts, the upper and lower half, to explain the description of the carving process. There are two walls where the inner wall was set up as the initial side to work into. It started from the lower half-edge to the middle arch (Fig 7A). Before resuming the strikes, the artisan rotated the piece 90° anticlockwise and finished the inner wall. Similar repetitions were applied to arrange the outer wall. A minor chisel rotation configured for upper and lower edges (Fig 7B). In general, the chisel was maintained at 45°. Moreover, the artisan lightly moved the chisel to enlarge the angle and shifted towards the extreme corner to obtain profound edges.

#### The letter E (Capital).

The letter E consists of a stem and three arms (upper, middle, and lower). It was carved on a slab of white marble using a flat chisel and a rounded wooden hammer. The artisan sketched the letter design beforehand and then started the strikes, making in this case a square groove: this type of groove is more rare than the more common ‘V’ and ‘U’ shaped: for a case study see the funerary inscription (9th century: A.D. 853) found during excavations in the foundations of the portico of the church of S. Maria in via Lata (Rome) [40,41].

A flat surface appears as the result of using the whole flat part of the chisel (S4 Video; S4 Text).

**Fig 8:**
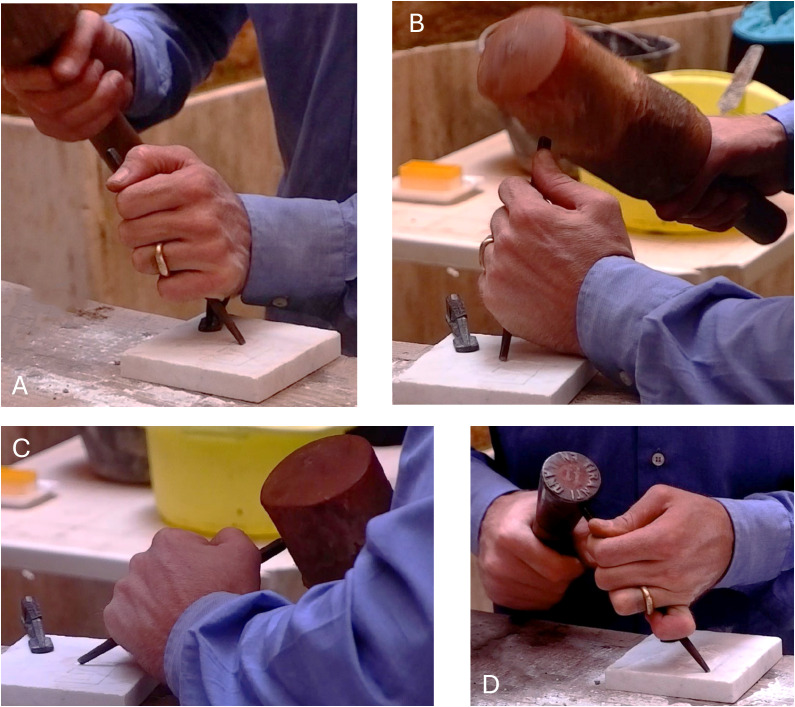
Stages of crafting the letter E (Capital) by the artisan. A: shows the starting point of the engraving from the lower arm, keeping a 60° angle; B and C: show how the edge of the lower arm was refined by rotating the chisel and working at different angles (from 45° to 90°); D: shows the switch from a wooden hammer to one with a rounded metal head.

The edge of the lower arm was the starting point of the carving. The artisan tried to maintain the angle of strike at 60° with continuous beats to complete the lower part of the stem (Fig 8A). He also made a repetition in the opposite direction. Moreover, he refined the lower arm’s border (including the edge) by turning the chisel towards the pointed corner that was in contact with the stone, working at various angles (45° to 90°) (Fig 8B–C).

Another part that has been examined is the intersection between the upper arm and the upper part of the stem. However, at some moments after the strikes on the stem started, he changed the hammer and finished the entire carving process using a rounded metal head hammer (Fig 8D). Stem produced by two upward strikes from the lower to the upper part. Then, the upper arm worked after performing sequences of horizontal direction outwards.

#### The letter E (Gothic).

The letter E of the Gothic era style developed using a flat chisel struck by a rounded metal head hammer. The letter has many distinguishing features compared to the previous E. A single arch substituted the stem, upper arm, and lower arm. Moreover, it has the middle arm in between and another vertical line that passes through the edge of those three arms.

This letter has a V-shape groove constructed by inner and outer walls. After shaping the vertical line, the artisan began to establish the arch. Additional strikes were recognized at the connection between both edges of the arch and vertical line, embodied as the broader end of the upper and lower arms (S5 Video; S5 Text).

**Fig 9:**
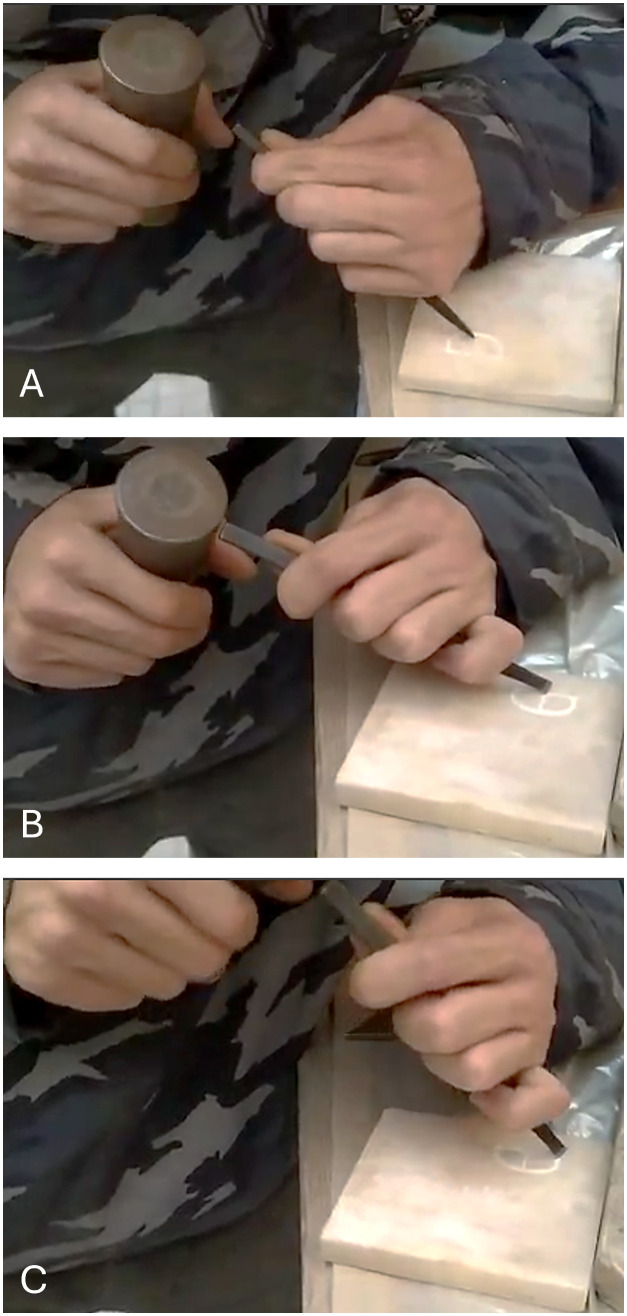
Stages of crafting the letter E (Gothic) by the artisan. The figures show the starting point of the engraving at the junction between the vertical line and the curve of the lower arm, with the chisel held at a 45° angle. The artisan then continued with the central arm before engraving the vertical arm, first working on the lower part and then, after rotating the object, the upper part.

The artisan focused on carving the inner wall of the arch with constant strikes upward. The starting point was between the joint of the vertical line and the curve of the lower arm. He maintained the chisel at 45° and repeated a similar procedure for the outer wall (Fig 9A). Then, the piece rotated 90° anticlockwise and the work emphasized towards the B part from the middle of the arch for both walls. An identical motion was attempted for the other side noticeably to construct the inner wall. In this position the artisan also shaped the middle arm; with two walls from the arch side upwards using the chisel tip at the beginning to give an impression of depth (Fig 9B). There was another rotation, and the strikes aimed to reform the outer wall of the arch upwards. At last, the artisan refined the edge of the upper and lower arm (Fig 9C).

### 3.2. The technological traces on the replicas

Table 1 describes the variables involved in the stone carving procedure documented during the reproduction of the letters: 1- raw material (type of stone, its surface topography); 2 – morphology of the support; 3 – activity, the action carried out, the direction of the incision with respect to the major axis of the letter; 4 – raw material the active hammer was made of, type of passive tool (tool tip morphology, area of the tip involved, tilting of the tip).

**Table 1:** Variables involved in the stone carving process, documented during the reproduction of the experimental letters.

Letters	Raw material type	Topography	Support type	Area	Activity	Action	Active tool (hammer)	Passive tool tip	Passive Tool Tip Size	Passive tool tilt	Part of the tip used	Enter Direction	Direction	Groove type
C	Peperino	Irregular	Slab	A – end	Inscription	Incision	Metal head	Flat tip chisel	1 cm	45°	Corner	Mixed	Mixed	V-shaped groove
C	Peperino	Irregular	Slab	B – body	Inscription	Incision	Metal head	Flat tip chisel	1 cm	45°	Corner	Vertical	Mixed	V-shaped groove
E (Gothic)	Pink marble	Regular	Slab	A – body	Inscription	Incision	Metal head	Flat tip chisel	1 cm	45°	Corner	Vertical	Vertical down-up	V-shaped groove
E (Gothic)	Pink marble	Regular	Slab	B – end	Inscription	Incision	Metal head	Flat tip chisel	1 cm	45°	Corner	Vertical	Vertical up-down	V-shaped groove
E (Gothic)	Pink marble	Regular	Slab	C – body	Inscription	Incision	Metal head	Flat tip chisel	1 cm	45°	Corner	Horizontal	Vertical down-up	V-shaped groove
E (Capital)	White marble	Regular	Slab	A – end	Inscription	Incision	Wooden head	Flat tip chisel	0,7 cm	45°	Corner	Oblique	Mixed	Square groove
E (Capital)	White marble	Regular	Slab	B – body	Inscription	Incision	Metal head	Flat tip chisel	0.7 cm	45°	Flat	Mixed	Vertical down-up	Square groove
R	White marble	Regular	Slab	A – body	Inscription	Incision	Wooden head	Flat tip chisel	1 cm	45°	Corner	Vertical	Vertical down-up	V-shaped groove
R	White marble	Irregular	Slab	B – end	Inscription	Incision	Wooden head	Flat tip chisel	1 cm	45°	Corner	Vertical	Vertical down-up	V-shaped groove
R	White marble	Regular	Slab	C – body	Inscription	Incision	Wooden head	Flat tip chisel	1 cm	45°	Corner	Vertical	Vertical down-up	V-shaped groove
R	White marble	Regular	Slab	D – body	Inscription	Incision	Wooden head	Flat tip chisel	1 cm	45°	Corner	Vertical	Vertical down-up	V-shaped groove
S	Grey marble	Regular	Slab	A – end	Inscription	Incision	Metal head	Round chisel (unghietto)	0,45 cm	60°	Corner	Oblique	Mixed	U-shaped groove
S	Grey marble	Regular	Slab	B – body	Inscription	Incision	Metal head	Round chisel (unghietto)	0,45 cm	60°	Flat	Vertical	Vertical down-up	U-shaped groove

Regarding the raw material, as documented in the experimental archaeology session, it plays a major role in the achievement of a clean incision and, consequently, in the achievement of a clear technological trace. Marble, the raw material most employed for epigraphs in the Roman era, has a fine, homogeneous microcrystalline texture lacking on inclusions. This composition facilitates the control of the motion and, therefore, facilitates a clean result. Conversely, *peperino*, a type of tuff widely diffuse during Middle Age in Central Italy, has a coarse and heterogeneous texture, frequently characterized by components of different size and hardness that do not facilitate the control of the motion and the resulting incision.

Regarding the activity and action carried out, the direction of the movement during the incision is a variable related both to the reciprocal position of the artisan body and the stone slab and the technique put in place by the artisan to carve the letters. Thus, it was of interest to document this variable.

Regarding the tools used during the experimental session, the documentation of different traces when using different chisels, distinguishable by their flat or round tip, is of great importance to infer the type of tool used by the artisans of the past. Likewise, technological traces documenting the area of the tip used (the entire tip or the corner) and the inclination of the tool during the activity are essential to infer the technique put in place by the artisans.

**Fig 10:**
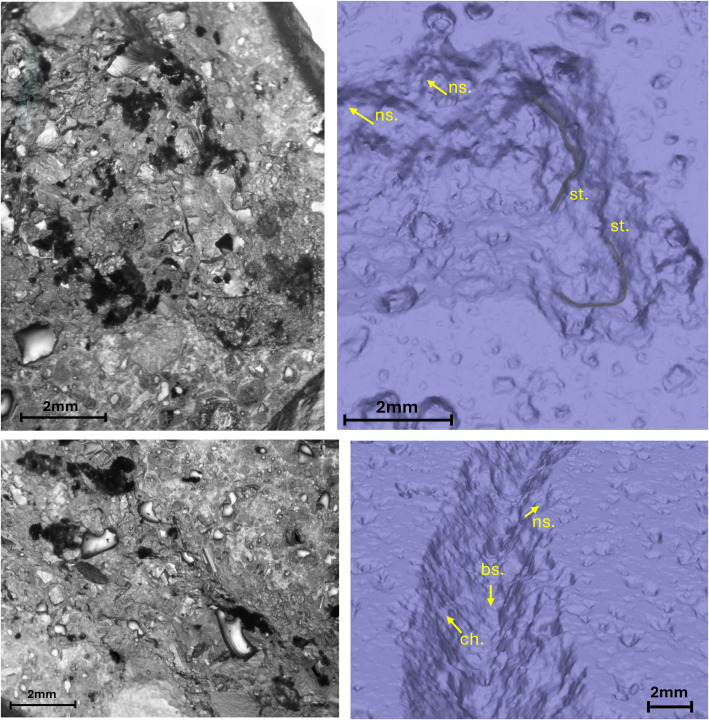
Technological traces of the letter C. The figure shows the selected areas (see Fig 3). The traces are not recognizable in the right-side view through the stereomicroscope in transmitted light due to the heterogeneity and roughness of the stone surface. The 3D mesh model on the left shows the traces marked by labels and arrows: Ns. Negative scars, Ch. Chatter marks, Bs. Base engraving. The darker and shaded areas indicate the steps (St.).

The comparison between the three methods of observation of the traces, by stereomicroscope in transmitted light of the resin, by stereomicroscope on reflected light of the stone surface, by micro-photogrammetry (S1 Fig) allowed to verify that the technological traces are better visible by resin or micro-photogrammetry except for the replica of letter incised on peperino. In this latter case, the heterogeneity and coarseness of the stone surface originated a poor reflection that prevented to recognize many of the technological traces at the stereomicroscope observation. Only the 3D model acquired by micro-photogrammetry allowed to detect them (Fig 10).

For each replica of letter, segments of the incisions exemplifying the end and the body were selected to document the technological traces (Fig 3). The analysis of different inscriptions and the experience gained have allowed us to develop a series of choices regarding the areas to be analysed, which could become standard in the structuring of a protocol. This is because the parts where the most important information can be found are those at the beginning and end of the engraving process, areas where overlaps occur, where there is a possible change in direction, and parts that curve.

For each segment, two variables related to the morphology of the carving tool tip were documented: the morphology of the incision in cross-section and the presence or absence of a secondary incision of the base were documented. The cross-section of the incision is essential for recognizing the morphology of the tip of the chisel and the tilting of the chisel during the activity. The secondary incision of the base is the “fingerprint” of the use of the corner of the flat tipped chisel tilted 45°.

Traces pertaining to the fatigue wear category [12,42] (chatter marks, negative scars and steps) and the abrasive wear category (striations) were detected on the walls and the base of each incision.

The orientation of the chatter marks and the scars allows documenting the direction of the motion; the presence of steps indicates the stop of the engraving motion, generally due to a change of direction. Striations are useful to detail the movement of the tool, distinguishing between rectilinear and circular direction.

**Fig 11:**
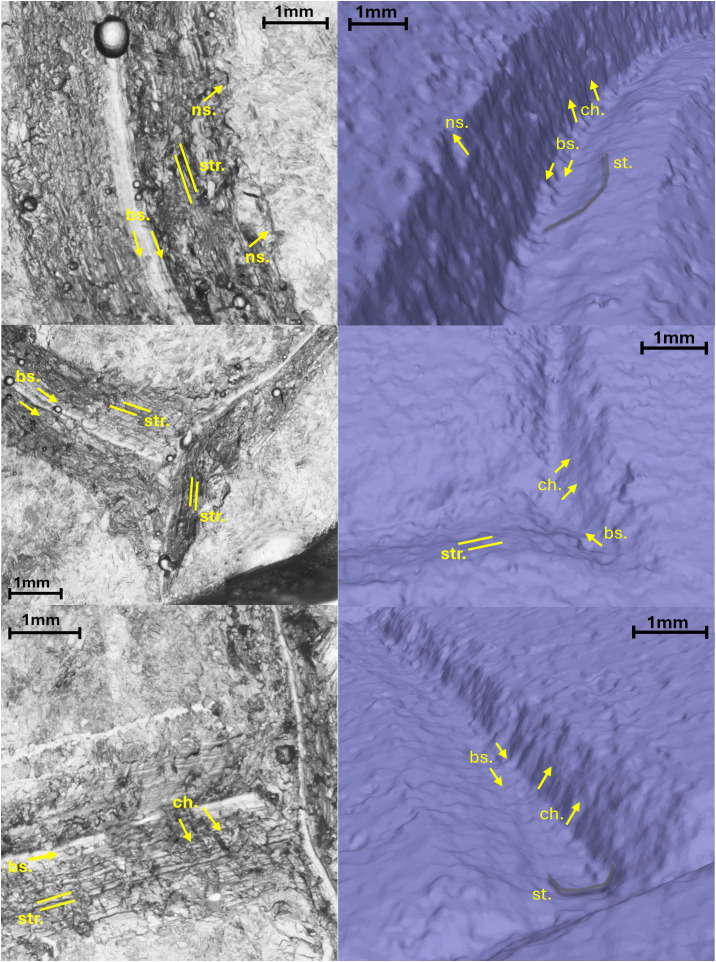
Technological traces of the letter E (Gothic). The figure shows the selected areas (see Fig 3). The traces are visible both in the right-side view through the stereomicroscope in transmitted light and in the 3D mesh model on the left. Chatter marks and steps are more visible in the 3D model, while the groups of striations are clearer under the optical microscope. The traces are marked with labels and arrows: Ns. (Negative scars), Ch. (Chatter marks), Bs. (Base engraving). The darker and shaded areas indicate the steps (St.). Parallel lines represent the groups of striations (Str.). The double arrow at the base indicates multiple passes.

**Fig 12:**
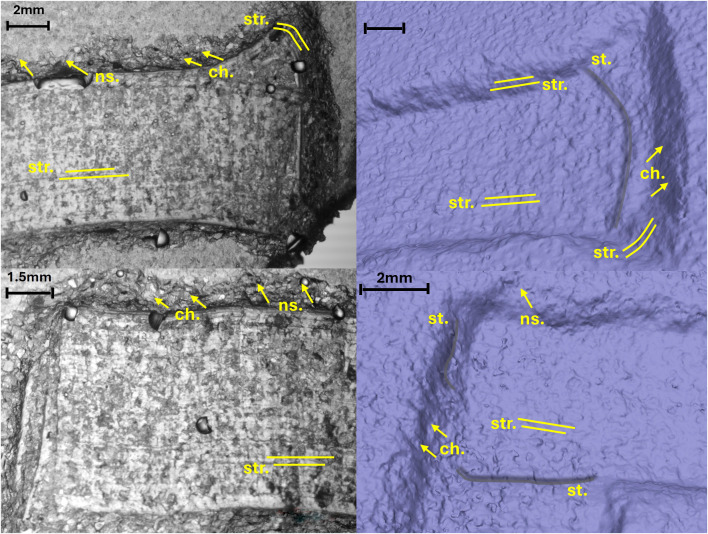
Technological traces of the letter E (Capital). The figure shows the selected areas (see Fig 3). The traces are visible both in the right-side view through the stereomicroscope with transmitted light and in the 3D mesh model on the left, except for the steps, which are only visible in the 3D model. The traces are marked with labels and arrows: Ns. (Negative scars) and Ch. (Chatter marks). The darker and shaded areas indicate the steps (St.). The parallel lines represent groups of striations (Str.). The base left by the tool’s tip is not present because the incision is square-shaped.

**Fig 13:**
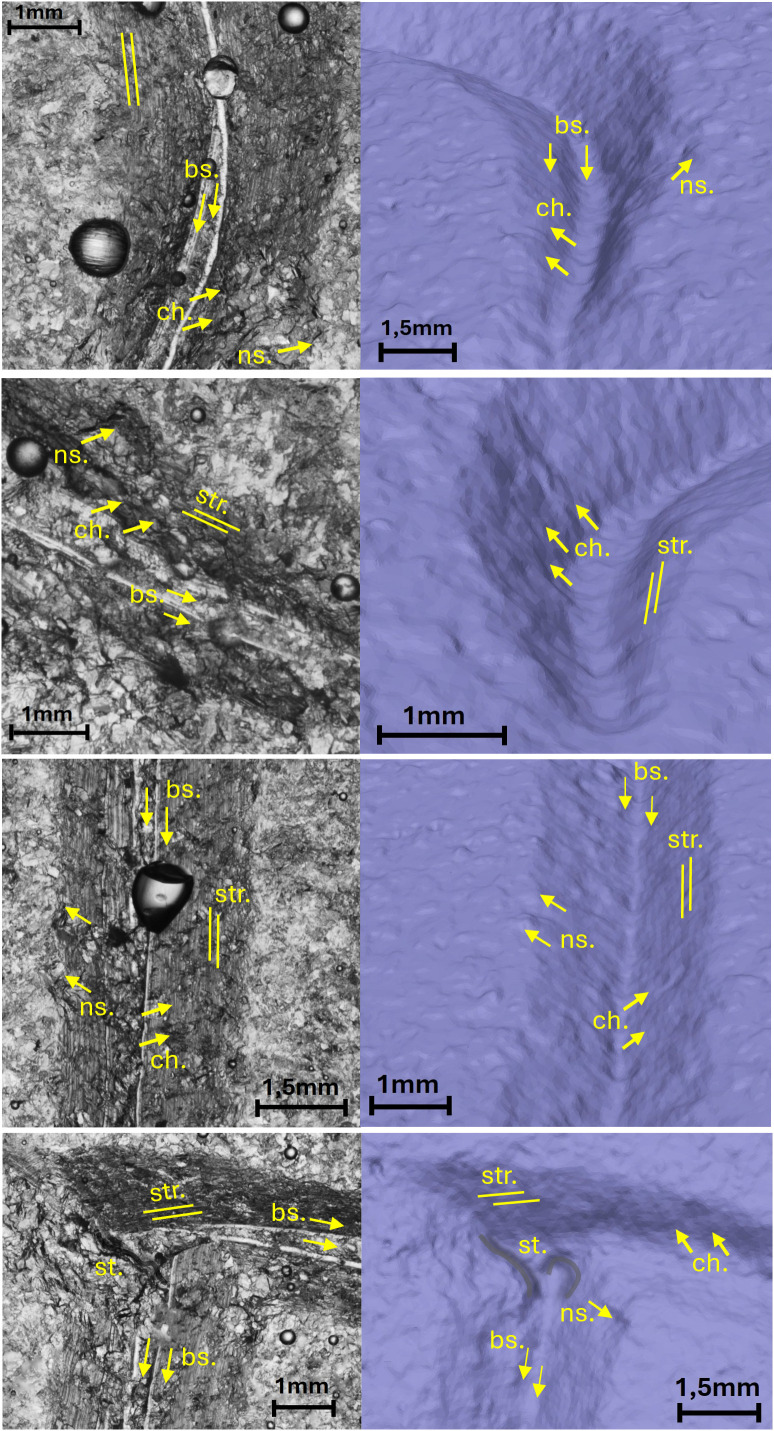
Technological traces of the letter R. The figure shows the selected areas (see Fig 3). The traces are visible both in the right-side view through the stereomicroscope in transmitted light and in the 3D mesh model on the left. Chatter marks and steps are more visible in the 3D model, while the groups of striations are clearer under the optical microscope. The traces are marked with labels and arrows: Ns. (Negative scars), Ch. (Chatter marks), Bs. (Base engraving). The darker and shaded areas indicate the steps (St.). Parallel lines represent the groups of striations (Str.). The double arrow at the base indicates multiple passes.

**Fig 14:**
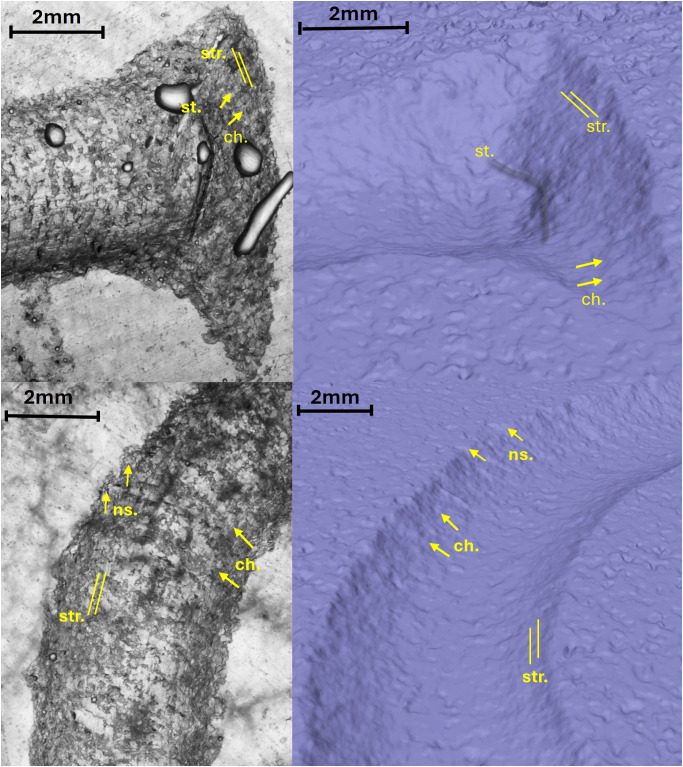
Technological traces of the letter S. The figure shows the selected areas (see Fig 3). The traces are visible both in the right-side view through the stereomicroscope with transmitted light and in the 3D mesh model on the left, except for the steps, which are only visible in the 3D model. The traces are marked with labels and arrows: Ns. (Negative scars) and Ch. (Chatter marks), which in this case are curved rather than rectilinear. The darker and shaded areas indicate the steps (St.). The parallel lines represent groups of striations (Str.). The base left by the tool’s tip is not present because the incision is U-shaped.

Below we present Table 2 documenting the variables used during the experiments (tools and gestures) and the resulting technological traces. The pictures (Figs 10–14) show selected areas of the letters with traces documented through optical methods and micro-photogrammetry.

**Table 2:** Documentation of technological traces linked to the variables (tools and gestures) observed in Table 1 during the experiments.

Letters	Area	Fractures/Cracks	Fractures/Cracks2	Fractures/Cracks3	Base Engraving	Group of striations	Shape of striations	Orientation of striations
C	A – end	Step	Negative scars	No	Yes	No	No	No
C	B – body	Rectilinear Chatter marks	Negative scars	No	Yes	No	No	No
E (Gothic)	A – body	Rectilinear Chatter marks	Negative scars	Step	Yes – double	Yes	Curved	Parallel
E (Gothic)	B – end	Rectilinear Chatter marks	No	No	Yes	Yes	Curved + Rectilinear	Mixed
E (Gothic)	C – body	Rectilinear Chatter marks	Negative scars	Step	Yes – double	Yes	Rectilinear	Parallel
E (Capital)	A – end	Rectilinear Chatter marks	Step	No	Yes	Yes	Curved + Rectilinear	Mixed
E (Capital)	B – body	Rectilinear Chatter marks	Step	Negative scars	No	Yes	Rectilinear	Perpendicular
R	A – body	Rectilinear Chatter marks	Negative scars	No	Yes – double	Yes	Curved	Parallel
R	B – end	Rectilinear Chatter marks	Negative scars	No	Yes – double	Yes	Curved	Parallel
R	C – body	Rectilinear Chatter marks	Negative scars	No	Yes	Yes	Rectilinear	Parallel
R	D – body	Rectilinear Chatter marks	Negative scars	Step	Yes – double	Yes	Curved + Rectilinear	Mixed
S	A – end	Rectilinear Chatter marks	Step	No	No	Yes	Rectilinear	Mixed
S	B – body	Curved Chatter marks	Negative scars	No	No	Yes	Curved	Parallel

**Fig 15:**
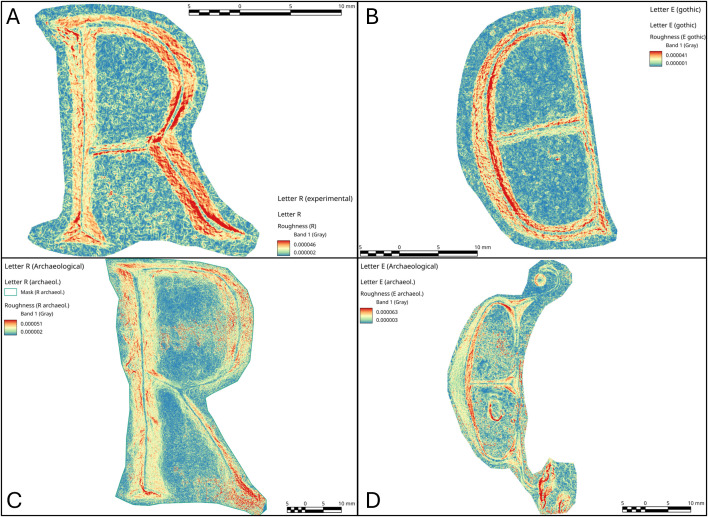
Surface roughness analysis. Comparison of the roughness of the experimental letters R (A) and E (Gothic) (B) with that of the archaeological letters R (C) and E (Gothic) (D).

The documentation of the surface roughness allowed us to identify areas of the letters that exhibit different levels of craftsmanship, smoothing, and levelling. Typically, the less rough areas correspond to the vertical strokes of the letters, where the artisan appears to have encountered fewer difficulties in execution. The most complex points are those where the stokes changes direction or along rounded ends (S2 Fig). An example is the letter E (Gothic) where the roughness is likely linked to the speed at which it was executed by the artisan (Fig 15B).

To synthesize the technological traces observed on the replicas of the letters and their significance we can point out:

1. The comparison between the traces documented with stereomicroscope modes and with micro-photogrammetry demonstrates that this latter technique acquired the whole traces perceivable with the optical modality; even better, in the case of the letter C incised in the coarse matrix of peperino stone, micro-photogrammetry is the only way to visualize the technological traces in their entirety (Fig 10);
2. The morphology and the inclination of the tool tip is detectable through the cross-section of the groove combined with the presence/absence of the incision of the base;
3. The direction of the incision (that is the last direction if the groove was produced with a back-and-forth direction, overlapping and delating the previous incisions) is detectable through the orientation of the chatter marks and negative scars;
4. The eventual back and forth directions are highlighted by a double or multiple base incision that, possibly, indicates also the slight change of the point of contact between the tool tip and the worked surface (Figs 11 and 13);
5. The perception of the direction of the incision and the change of this direction is enhanced by the observation of the striations affecting the walls of the groove;
6. Especially in the approaching the end of the letter, striations document the change of the direction of the gesture of the artisan in preparation for the movements involved in production of the end section (Figs 11 and 12);
7. The presence of steps inside the groove testifies to an abrupt change of depth when incisions with opposite directions come into contact during the production of the body of the letter or when the artisan starts the production of the end. In this latter case, the step testifies the change of the depth in the point where the incision turns from the body to the starting point of the end (Figs 12–14).

Slope analysis proved to be very efficient in confirming the presence of the traces qualitatively observed during the optical and micro-photogrammetry inspection. In particular, chatter marks and striations were clearly documented by the sections of the slope taken at various points of the body and the ends (S3, S4 and S5 Figs). To have a direct comparison with the archaeological letters, we chose to focus on the letters R and E (Gothic).

**Fig 16:**
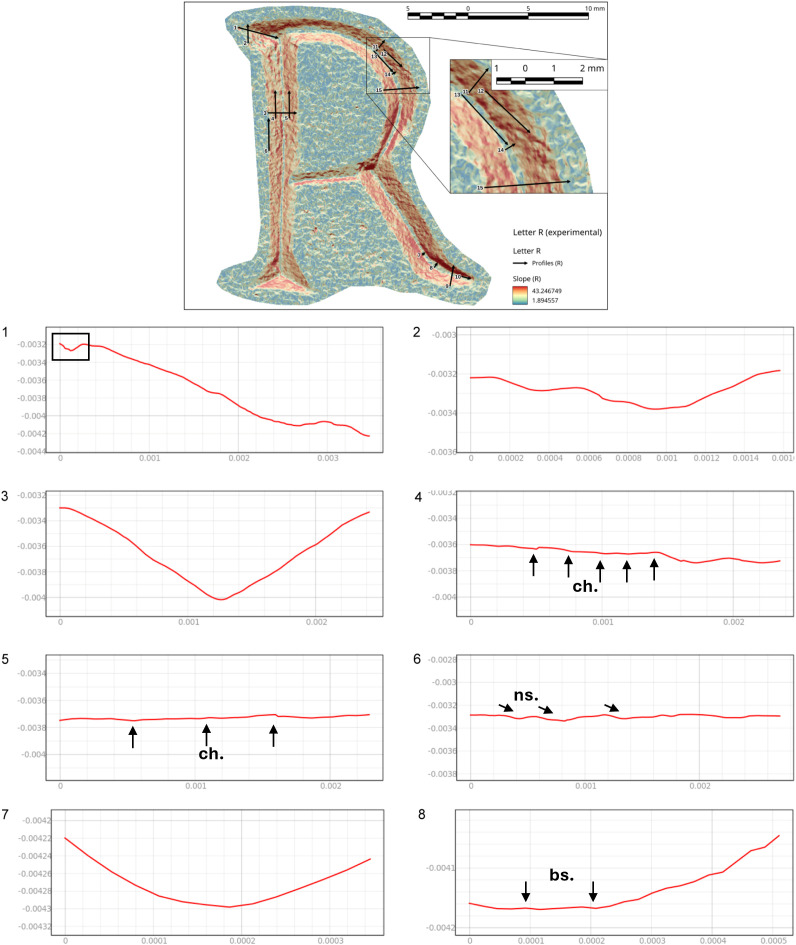
Slope analysis of the experimental letter R with profile extrapolation at the marked points (from point 1 to point 8). The profile of the traces at different points is marked with labels and arrows: Ns. (Negative scars), Ch. (Chatter marks), Bs. (Base engraving).

**Fig 17:**
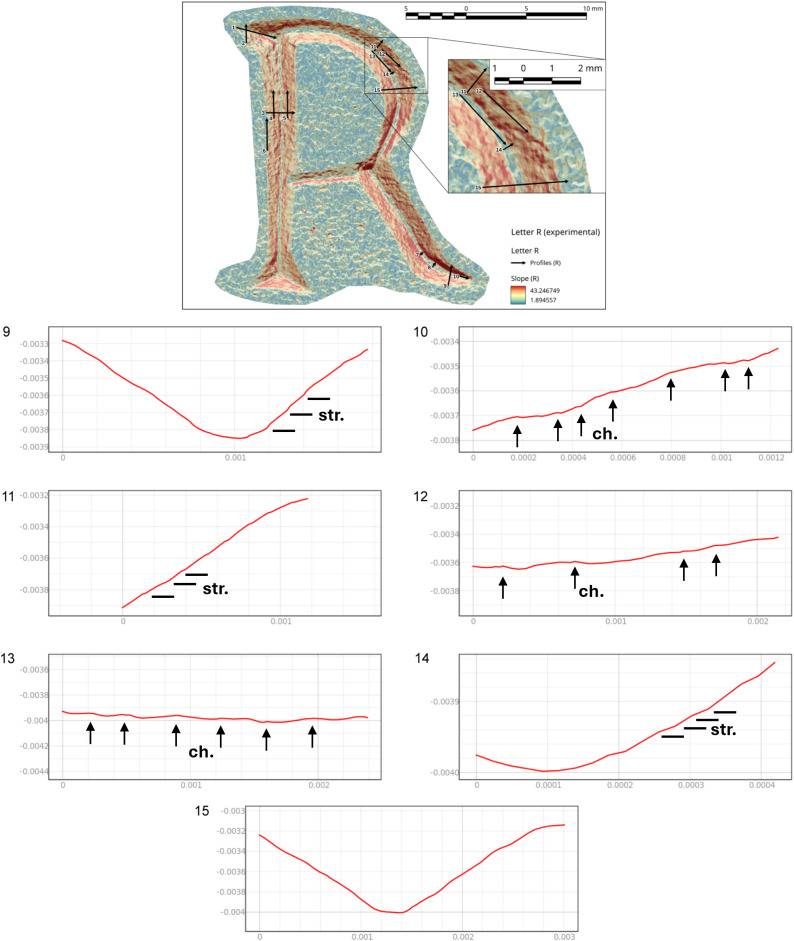
Slope analysis of the experimental letter R with profile extrapolation at the marked points (from point 9 to point 15). The profile of the traces at different points is marked with labels and arrows: Ch. (Chatter marks) and the straight lines correspond to groups of striations.

**Fig 18:**
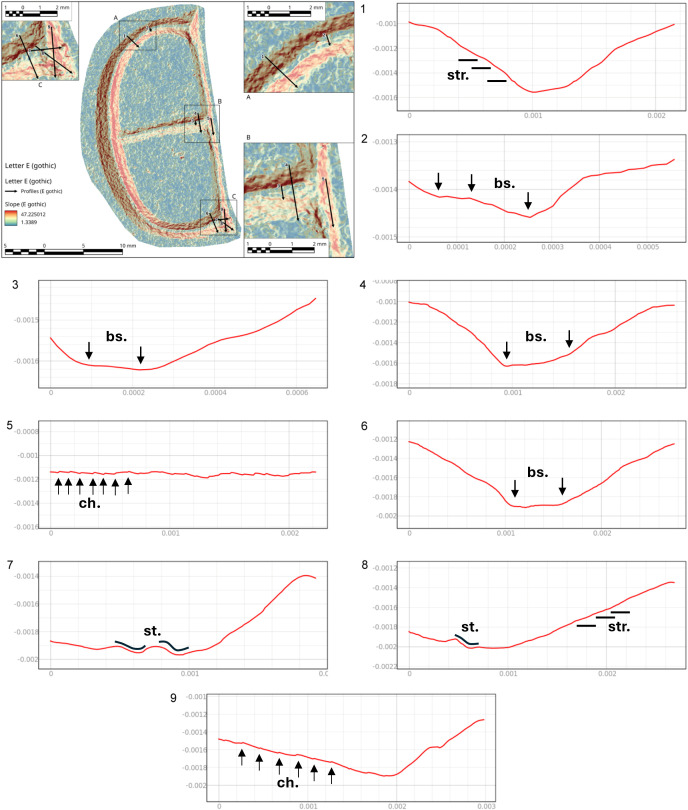
Slope analysis of the experimental letter E (Gothic) with profile extrapolation at the marked points. The profile of the traces at different points is marked with labels and arrows: Ch. (Chatter marks) and Bs. (Base engraving). The straight lines correspond to groups of striations (str.), while the curved lines correspond to steps (st.).

In the case of the letter R, the analysis was also useful in identifying the entry point of the chisel (Fig 16 point 1). The high regularity of the walls and base in the execution of the V-shaped incision (Fig 16 points 3 and Fig 17 points 9 and 15) is particularly evident in the stem. This part is usually easier for the artisan to create because the incision consists of a straight line without changes in direction. Comparing point 3 and point 15 (Figs 16 and 17), it becomes clear that the latter has a wider wall than the other. This characteristic appears more frequently in curved sections and is likely influenced by the artisan’s range of motion, the size of the incised surface, and the type of gestures involved. Moreover, the base section on the slopes of the E (Gothic) and the R clearly shows multiple passes of the tool’s tip, which are even more visible and identifiable than those seen on the 3D model mesh (Fig 16 point 8 and Fig 18 points 2–4, 6). The cross-section view also shows groups of striations, which are slightly visible along the walls of the incision (Fig 17 points 9, 11, 14 and Fig 18 points 1, 8).

The longitudinal section, on the other hand, highlights the presence of chatter marks, which, in the case of the letter R, appear highly regular in both spacing and morphology, affecting both the base (Fig 17 points 10 and 13) and the walls (Fig 17 point 12). This allows for a quantitative comparison between the two mirrored surfaces (Fig 16 points 4 and 5). The chatter marks of the E (Gothic) appear very close to each other, likely due to the speed of execution and the small size of the letter. They are also sharply defined on the stone surface (Fig 18 points 5 and 9), suggesting that the tool passed over the area only briefly. This is also evident from the surface roughness (Fig 15A), indicating minimal smoothing of the surface.

Finally, though to a lesser extent, negative scars are also visible, for example at point 6 of the letter R (Fig 16), where depth can be analysed, although the direction of the gesture cannot be determined from this alone; instead, it emerges from the combination of qualitative variables. In the slope of the E (Gothic) (Fig 18 points 7 and 8), steps are also visible, highlighting both depth and morphology. This gives us an idea of how deep the tool penetrates at certain points, especially in the creation of the final parts of the letter.

### 3.3. The technological traces on the archaeological epigraphs

Table 3 shows all the data related to the study of the sampled archaeological epigraphs. The first part of the table outlines the information we can obtain from the historical reconstruction and the context of the inscription:

**Table 3:** Documentation of technological traces on archaeological letters, providing information about the type of tool and the gesture.

Site	Letters	Raw material type	Topography	Support type	Area	State of preservation	Activity	Action	Fracture/ Cracks	Fracture/Cracks 2	Fracture/Cracks 3	Base Engraving	Group of striations	Shape of striations	Orientation of striations	Inferred passive tool tip	Inferred part of the tip used	Groove type	Inferred incision enter direction	Inferred incision direction
Viterbo Civic Museum	E (Gothic)	White Marble	Regular	Slab	A – body	Restoration	Inscription production	Incision	Rectilinear Chatter marks	Negative scars	No	Yes	Yes	Rectilinear	Parallel	Flat tip chisel	Corner	V-shaped groove	Vertical	Vertical down-up
Viterbo Civic Museum	E (Gothic)	White Marble	Regular	Slab	B - end	Restoration	Inscription production	Incision	Rectilinear Chatter marks	Convex Chatter marks	No	Yes	Yes	Curved + Rectilinear	Mixed	Flat tip chisel + Round chisel (unghietto)	Corner	V-shaped groove	Undetermined	Undetermined
Cencelle Medieval town	R	White Marble	Regular	Slab	A – body	Altered	Inscription production	Incision	Rectilinear Chatter marks	No	No	Yes	Yes	Rectilinear	Parallel	Flat tip chisel	Corner	V-shaped groove	Undetermined	Vertical up-down
Cencelle Medieval town	R	White Marble	Regular	Slab	B - end	Altered	Inscription production	Incision	Rectilinear Chatter marks	Step	No	Yes	Yes	Curved + Rectilinear	Mixed	Flat tip chisel	Corner	V-shaped groove	Oblique	Vertical down-up

1. Details about the site of origin, the conservation context, the specific letter chosen from the entire inscription, and the parts of the letter examined (letter, location, site, area);
2. Details concerning the materiality of the epigraphs that is, the type of raw material and its surface topography; the morphology of the support (type); the state of conservation, including any restoration actions and the presence of surface alterations or degradation; the general activity and general action involved in carving the letter.

The second part focuses on the information inferred from technological traces analysis coupled with roughness and slope analysis:

1. The type of traces, divided in the two categories of fatigue wear and abrasive wear [12,42];
2. The inferred type of tool used and the portion of tip used;
3. The inferred general direction of the movement, the entry direction in specific areas, the groove type, allowing for an interpretation of the gesture, the type of incision, and the possible steps taken by the artisan.

**Fig 19:**
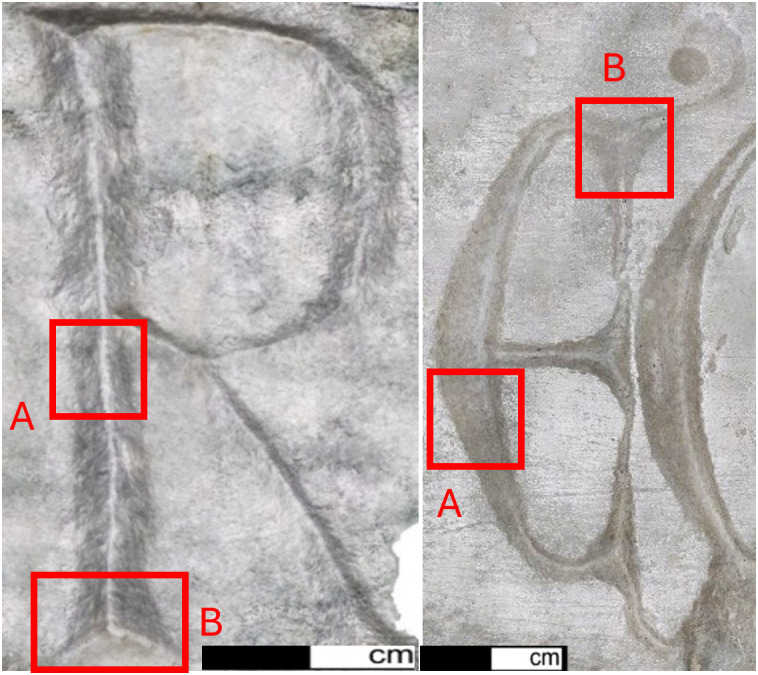
Archaeological documentation: letters R and E (Gothic). Selection of the analyzed portions.

An important factor not to be underestimated is the presence, in most cases, of alteration and degradation layers on the surface, which can interfere with the accurate observation of the traces and alter their morphology. In addition to post-depositional processes related to the artifact’s preservation over time, there are also restoration interventions carried out over the years. These are often dated to the 1900s, a period when the focus was more on the aesthetics and reconstruction of the object rather than its proper conservation. The methods and tools used during that time were often invasive, leaving significant marks – sometimes visible to the naked eye and, in other cases, identifiable through mesoscopic-level documentation. In this case, it was possible to observe, through photos taken with the portable digital microscope, that the epigraph from Viterbo had a white encrustation within the letter incisions, present only in certain areas. The external surface of the epigraph was very shiny, showing evidence of polishing and surface treatment, likely performed with restoration pastes. Possibly, these substances also seeped into the incisions originating these encrustations. While the external surface was cleaned and prepared for museum display, the material left inside the incisions, and thus not visible, was not removed. Fortunately, this encrustation was only present in some parts of the letters. When selecting areas for detailed analysis, efforts were made to avoid sections where the white substance was more concentrated. The epigraph from Cencelle, on the other hand, shows slight surface alteration, like a smoothing, which did not interfere with the observation of the traces. As with the experimental letters, in this case too, two segments of the incisions were selected for each inscription to document the technological traces, represented by a portion of the body and the end part of the letter (Fig 19).

**Fig 20:**
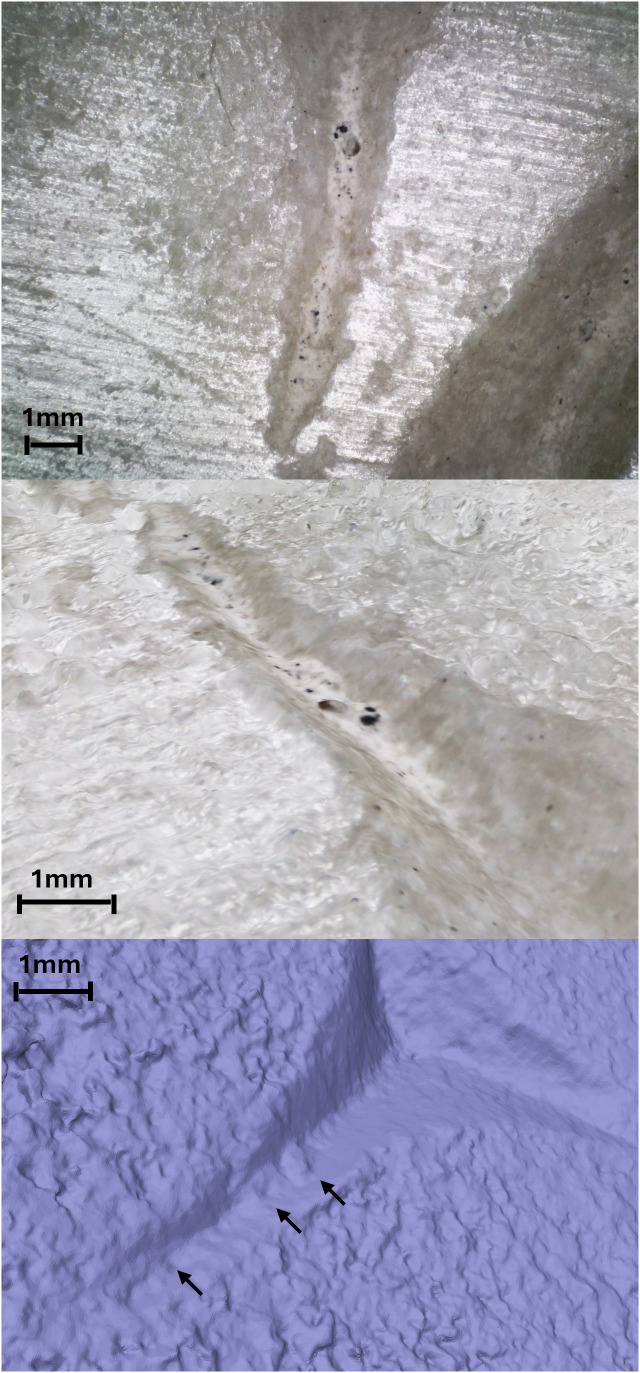
Restoration interventions on the archaeological letter E (Gothic). At the top, a digital microscope photo shows a white encrustation within the letter’s incisions and a very shiny surface on the outside, showing evidence of polishing and surface treatment. In the center, the 3D model texture allows for measuring the thickness and width of the encrustation. At the bottom, a view of the 3D model’s mesh with arrows indicating the morphology of the encrustation, which covers the traces.

Being non-portable, documentation was conducted at the location where the epigraphs were preserved, using only micro-photogrammetry approach. In both cases, it was not possible to use two-component silicone to mould the incisions because, based on test trials on white marble, it can leave highly visible oil marks. Additionally, since both artifacts are intended for museum display, this type of sampling was not permitted. The observation of the 3D mesh obtained from the micro-photogrammetry allowed us to identify the added layer of encrustations resulting from restoration, measure its width and thickness, and distinguish it from the original surface. This helped evaluate the accuracy of the data and make better-informed decisions on which parts to analyse. This further confirms the potential of micro-photogrammetry for an initial assessment of surface alterations and degradation (Fig 20).

Traces pertaining to the fatigue wear category [12,42] (chatter marks, negative scars and steps) and the abrasive wear category (group of striations) were detected on the walls of each incision.

Thanks to the 3D visualization, it was possible to identify the morphology of the incision in cross-section and determine the tools used. The roughness and slope analysis provide information on the section profiles of the engraving, confirming the traces observed through qualitative analysis and highlighting their morphology, depth, and the frequency of marks on the analyzed surface.

It is important to emphasize how the workspace and type of gestures used for archaeological inscriptions, likely carved on large slabs placed either horizontally on the ground or already set vertically on a wall, differ significantly from those used for experimental epigraphy. The small slabs on which the experimental letters were carved could be rotated and moved as needed by the artisan, which influenced the regularity of the gesture.

The roughness analysis of both archaeological letters highlights areas that were less worked and left rougher, likely due to these challenges. The roughest parts are almost always the apices of the leg and stem, as well as the arches, where a circular movement was performed. In the letter R from Cencelle, there is evident difficulty in carving the end of the leg (Fig 15C), while in the E (Gothic) from Viterbo, the right wall of the central arch appears less worked and broader, likely due to the artisan’s position and working method. Furthermore, roughness analysis highlights areas where errors and imprecision of the artisan appear, particularly in the decorative elements of the E (Gothic) (Fig 15D).

#### The letter E (Gothic) from Viterbo epigraph.

**Fig 21:**
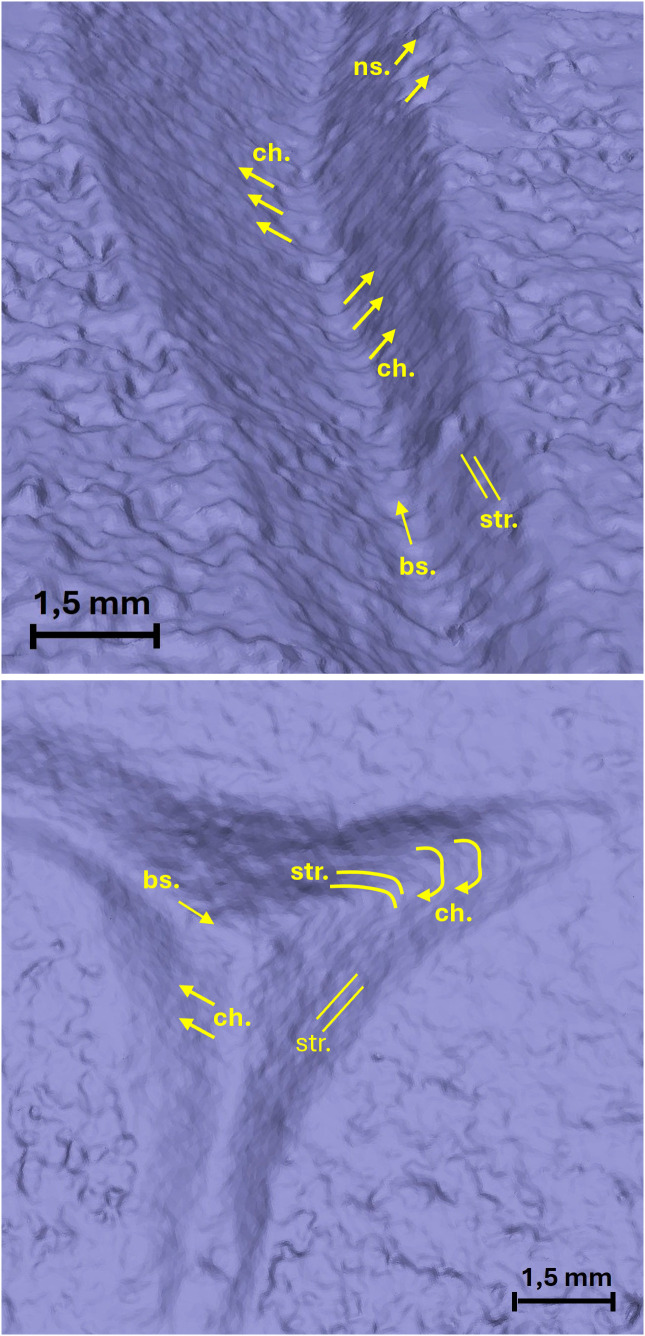
Technological traces of the archaeological letter E (Gothic). The figure shows the selected areas (see Fig 19). The traces are marked with labels and arrows: Ns. (Negative scars), Bs. (Base engraving) and Ch. (Chatter marks), which can be either rectilinear or convex. The rectilinear and curved lines represent the groups of striations (Str.).

The analysis of the letter E (Gothic) provides information regarding the use of two tools: a flat-tipped chisel for most of the letter and a round chisel for the ends and the filigree floral decorations. This conclusion is based on the morphology of the chatter marks, which are both rectilinear and convex, with the latter overlapping the former. Conversely, the chatter marks observed on the body section testify to the use of flat-tipped chisel only (Fig 21).

The base of the groove, in both the end and body sections, document the use of the corner of the chisel to produce a V-shaped groove morphology. It is not possible to determine the size of the tool’s tip because the width of the incision wall may not precisely correspond to the tool’s tip. This also depends on the number of times the incision was passed over, which could have expanded the incision to varying degrees (Fig 21).

The direction of movement is indicated by the chatter marks; particularly those visible on the right wall of the incision indicate a vertical movement pattern, from down-up according to the main axis of the letter and its orientation, in the body area. On the left wall, the chatter marks are less inclined and more frequent; one hypothesis – though not fully corroborated by the experimental data – is that this may reflect multiple passes with the tool in different directions to create the broader section of the letter (Fig 21).

In general, the engraving appears to be well-executed, showing no signs of errors or inaccuracies. However, near the decorative elements extending from the letter, particularly in the lower part, some difficulties can be observed in carving the circular cavities. Striations along the walls, left by a small drill, are visible, along with irregular incisions caused by the drill tip slipping. This section appears to be a mix of errors and attempted corrections of the decoration, which, however, were not successfully executed.

**Fig 22:**
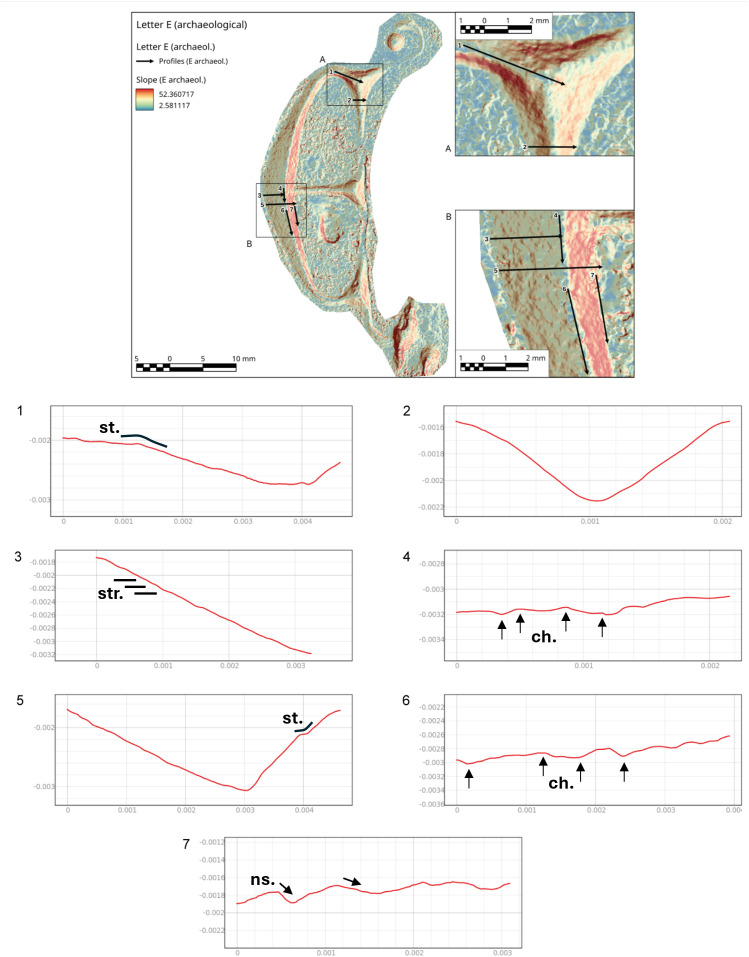
Slope analysis of the archaeological letter E (Gothic) with profile extrapolation at the marked points. The profile of the traces at different points is marked with labels and arrows: Ns. (Negative scars) and Ch. (Chatter marks). The rectilinear lines correspond to groups of striations (str.), while the curved lines correspond to steps (st.).

The slope analysis clearly highlights the steps formed at the end of the letter. These steps result from the artisan carving deeply at the letter’s endpoints to execute the engraving (Fig 22 point 1).

Examining the cross-section at different points of the letter reveals a precise and well-executed incision. The base maintains a V-shape without rounding, which typically occurs with multiple tool passes. Comparing the cross-section along the end of the letter (Fig 22 point 2) with that along the main arch (Fig 22 point 5) confirms what the roughness analysis already indicated: the right side of the stem is narrower and less precisely executed than the left. Small steps are visible along its surface, likely due to the greater difficulty of shaping the curved section and the artisan’s working position. The longitudinal profiles of the arch’s base reveal deep and irregular chatter marks (Fig 22 points 4 and 6). These marks suggest that only a few tool passes were made in this area, leaving the base rough and unfinished. Additionally, the 3D mesh does not show evidence of multiple tools passes along the base.

Finally, the profile at point 7 (Fig 22) highlights negative scars, clearly illustrating the tool’s direction during carving.

#### The letter R from Cencelle epigraph.

**Fig 23:**
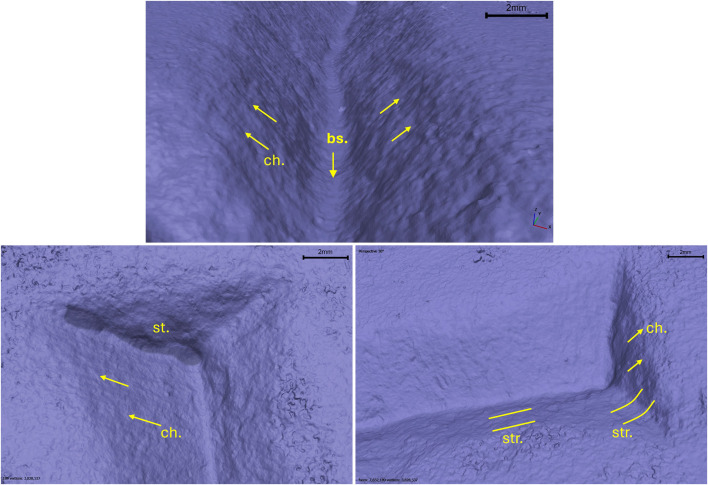
Technological traces of the archaeological letter R. The figure shows the selected areas (see Fig 19). The traces are marked with labels and arrows: Bs. (Base engraving) and Ch. (Chatter marks). The rectilinear and curved lines represent the groups of striations (Str.). The darker and shaded areas indicate the steps (St.).

The end of the letter R shows chatter marks with an oblique direction, suggesting a movement of the chisel from down to up to produce the end corner. The morphology of the chatter marks documents the use of a flat-tipped chisel. The base of the groove, in both the end and body sections, document the use of the corner of the chisel to produce a V-shaped groove morphology (Fig 23). Also in this case, it is not possible to determine the precise size of the tip of the chisel.

The presence of steps on the end section indicates the point where the tool stopped during the incision process, as well as an overlap caused by multiple passes or points where different movement directions intersected (Fig 23). It underlines difficulties in controlling the gestures to produce this part of the letter, documented also in the replicas. The redundance of the steps could document a possible low skill of the artisan or not accurate production.

In general, it is also possible to notice any errors or methods used in the creation of the inscription. Moreover, the letter from Cencelle has areas where the wall has partially collapsed and points of deviation in the incision, possibly due to the presence of quartz in the stone that was dislodged during the tool’s passage. Despite this, the areas where a loss of material have been somewhat repaired through multiple passes of the tool.

**Fig 24:**
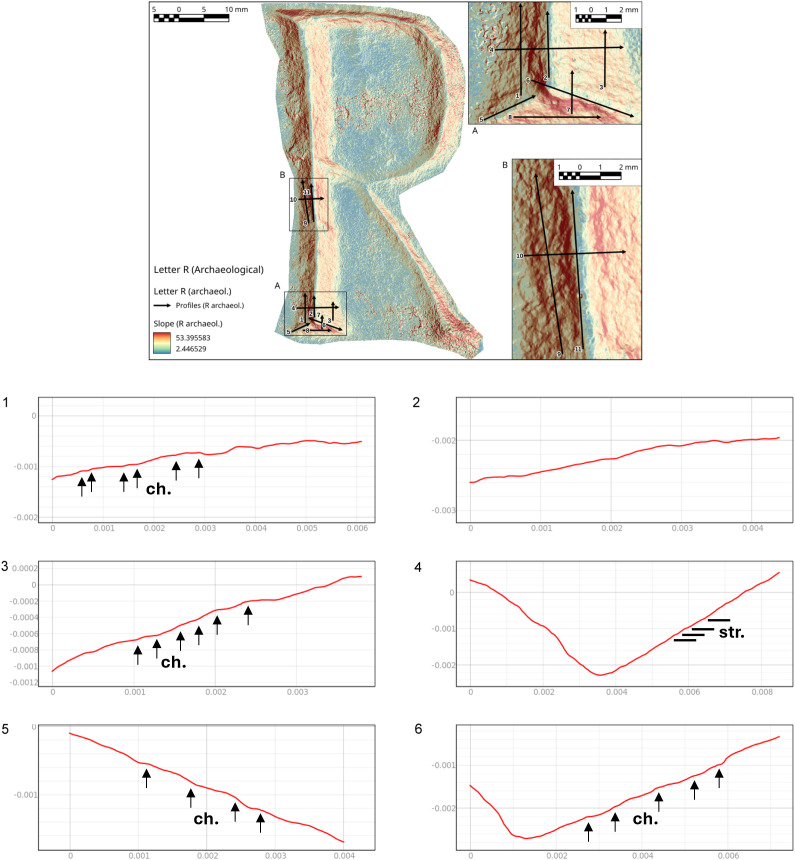
Slope analysis of the archaeological letter R with profile extrapolation at the marked points (from point 1 to point 6). The profile of the traces at different points is marked with labels and arrows, which represent the Chatter marks (Ch.). The rectilinear lines correspond to groups of striations (str.).

**Fig 25:**
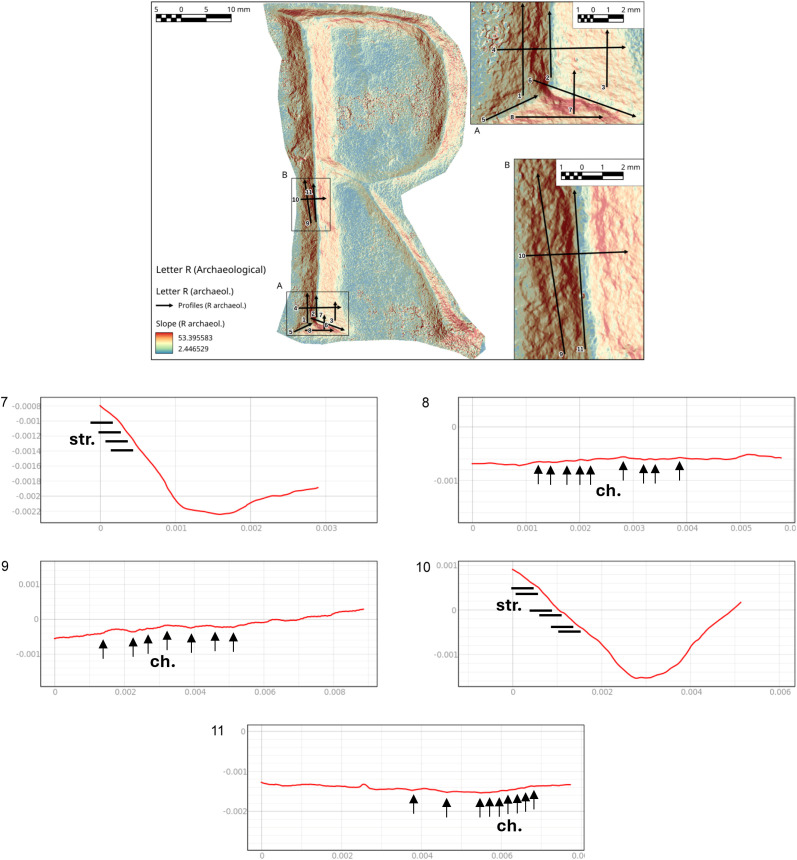
Slope analysis of the archaeological letter R with profile extrapolation at the marked points (from point 7 to point 11). The profile of the traces at different points is marked with labels and arrows, which represent the Chatter marks (Ch.). The rectilinear lines correspond to groups of striations (str.).

From the slope analysis, in which several transverse and longitudinal profiles were cut along the stem, chatter marks emerge as the most prominent feature. They appear very regular and well-defined, allowing for a comparison of their distances and recurrence (Fig 24 points 1, 3 and Fig 25 points 8, 9).

When compared to the experimental letter R (Fig 16 points 4–5 and Fig 17 points 10, 12, 13), the chatter marks appear more consistent, especially in their spacing, than in the archaeological letter. This is likely due to both the larger size of the archaeological letter and the multiple passes and levelling work performed by the early medieval artisan. Between the two walls of the stem, the right one appears more regular, as highlighted by the chatter marks (Fig 24 point 3), the roughness (Fig 15C), and the section at point 4 (Fig 24).

The chatter marks on the walls along the end part of the stem are also highly regular, though closer together (Fig 25 point 8), as the oblique gesture was executed more quickly due to the reduced surface area. The end of the stem is particularly well-made and does not display the usual defects or difficulties that often appear in the final parts of letters. The chatter marks at the base of the end part are less closely spaced and more regular (Fig 24 points 5 and 6) compared to those at the base of the stem, which are closer together and less distinct (Fig 25 point 11). This suggests that the chisel tip was passed multiple times along the stem.

Finally, in addition to the chatter marks, striations can be observed in the section, just as in the experimental letters (Fig 17 points 9, 11, 14). These striations are clearly visible and not compromised by potential surface alterations of the archaeological sample, further confirming the good condition and reliability of the analyzed surface (Fig 24 point 4 and Fig 25 points 7, 10).

## 4. Discussions

The proposed approach differs from previous attempts to study tool traces on inscriptions and to document incisions in several key aspects. In recent years, research on tool marks on stone inscriptions has gained increasing attention, particularly due to the intersection between epigraphic studies and rock art research. The introduction of visual archaeology techniques, such as photogrammetry and Reflectance Transformation Imaging (RTI), has significantly improved the documentation of eroded or poorly visible surfaces, enhancing the understanding and interpretation of epigraphic texts.

Despite these advancements, more detailed studies on incisions [11,23,24], have focused solely on the analysis of incision profiles, morphology, and surface characteristics. However, they have not been supported by experimental reproduction of incisions using different tools. This limitation prevents precise identification of the tools used and does not provide insights into the artisan’s gestures and skills. The development of an experimental reference collection is therefore essential, as comparing experimental and archaeological material allows for a better understanding of tool marks and the functional or technical reasons behind them. On the other hand, in the rare cases where experimental archaeology has been used to reconstruct epigraphic production processes [25,26], a detailed mesoscopic analysis of tool traces was not conducted, limiting the full potential of experimental approaches. This study therefore aims to emphasize the importance of an integrated approach between the two methodologies to promote the innovative nature of research in the epigraphic field, but above all to highlight the value of using experimental techniques to understand how certain practices and procedures were carried out. The traceological approach is also fundamental for identifying traces and using them to interpret archaeological data. Only in this way can we go beyond the stylistic and paleographic interpretation of inscriptions to gain detailed knowledge of aspects related to working techniques, gestures, and artisanal skills.

Another key innovation of this research is the application of micro-photogrammetry as an on-site documentation method. The main advantages of this technique include the portability of the equipment, low costs, and a non-invasive, non-destructive acquisition process. These factors are particularly important in epigraphy, where inscriptions are often immovable or located in hard-to-reach places. On the other hand, the use of laboratory digital 3D microscopes, often used for this type of analysis, while offering a high level of magnification, is not very practical when documentation must take place exclusively in situ. Moreover, for the type of traces analysed, observation at a mesoscopic scale is already optimal for highlighting the features and traceological variables necessary for the study. If compared to previous micro-photogrammetric tests [12], the addition in this case of a 6 cm protective cone to the digital microscope significantly improved the quality of detail capture in 3D models by reducing external light interference. Although this adjustment increased the scanning time, the resulting data quality was far superior. Additionally, optimizing the microscope settings by combining maximum polarization with LED lighting effectively mitigated reflections on bright surfaces.

An additional point of discussion arises from the comparison between three trace observation methods – stereomicroscopy in transmitted light on resin, stereomicroscopy in reflected light on stone, and micro-photogrammetry – showed that technological traces are best observed on resin or through micro-photogrammetry. Notably, micro-photogrammetry was able to capture all traces detectable through optical observation. In some cases, such as the letter C engraved in the coarse-grained *peperino* matrix, it was the only method capable of fully revealing the technological traces. This further validates the proposed technique, making it competitive with other trace documentation methods and particularly suitable for field applications.

The experiment conducted with an experienced artisan, where different letters were carved in various styles and materials, including peperino and marble, made us realize how much the raw material influences the stone-carving process. Depending on the letter to be carved, its size, the support, and the raw material, the artisan changed the type of chisel and, especially, the hammer (wood or metal). Observing the artisan’s work also led to reflections on the working method and space: it is essential to highlight the differences between the workspace and carving techniques used for archaeological inscriptions and those employed in experimental epigraphy. Inscriptions on large slabs, which were likely placed horizontally on the ground or mounted on a vertical surface, required different working conditions than the experimental setting. In this case, the small slabs used for carving the letters could be freely rotated and repositioned by the artisan, directly impacting the consistency of the carving gestures.

The traces identified on the surface allowed us to understand the morphology and angle of the chisel’s tip, as well as the direction of movement, tool passages across the surface, and possible interruptions due to changes in direction or difficulties during the sculpting process. Observing both experimental and archaeological material helped identify the key areas of the letters where most traces were found: at the beginning and end of the carving process, where overlapping, changes in direction, and complex curved or uniquely shaped areas typically occur.

Indeed, based on the micro-topographic analysis of roughness, the smoother areas typically correspond to the vertical strokes of the letters, where the artisan appeared to encounter fewer difficulties. The most complex areas are where the strokes change direction or along the rounded edges. A recurring feature highlighted by both micro-photogrammetry and slope analysis is the presence of one side of the incision being wider than the other, often seen in the curved sections. This is likely influenced by the artisan’s hand movement range, the size of the carved surface, and the type of gesture used. We can therefore say that the quantitative analyses of roughness and slope proved to be highly effective in confirming the presence of traces that were qualitatively observed during optical inspection and micro-photogrammetry. Besides making chatter marks, negative scars, and steps visible, striations were clearly documented, even in areas difficult to see through micro-photogrammetry, demonstrating its potential in detecting details not revealed by purely qualitative methods. The distance between the various traces and the depth reached, which can always be visualized through the profiles obtained from the slope analysis, also indicates the force applied to the tool, reflects the speed of execution, and may correlate with the reduced size of the carved letter.

The results from the analysis of archaeological inscriptions show that an experimental and traceological approach allows to identify differences and similarities in the technological production of inscriptions. In our study, we compared two letters: one from the 9th century (Early Medieval) and the other from the 13th century (Late Medieval), both from the same geographical context in northern Lazio and made from the same raw material. The traces that can thus be identified based on the knowledge acquired through traceological analysis from both the experimental and archaeological letters belong to two main categories: fatigue wear (chatter marks, negative scars, and steps) and abrasive wear (striations). The comparison between the archaeological and experimental traces revealed several important aspects. The first is related to the tools used. In both cases, a flat-tipped chisel was identified thanks to the straight chatter marks already observed in the experimental letters C, E (Gothic), and R. The chisel was used along the body of the letter and at the ends at a 45° angle. Additionally, the trace left at the base of the incision shows that only the chisel’s corner was used to create a V-shaped profile. In particular, the analysis of the archaeological letter E (Gothic) also showed the combined use of the flat-tipped chisel for most of the letter and a rounded-tipped chisel for the ends and decorative elements. This was visible thanks to the presence of convex chatter marks, as seen on the experimental letter S. The trace analysis also revealed information about the gestures and techniques. The general direction of movement is indicated again by the chatter marks and, in some places, also by the negative scars, as we were able to determine from observing the experimental traces. In the case of the E (Gothic), the movement was from down-up in a vertical motion, while for the R there was overlap caused by multiple passes or points where different movement directions intersected along the body of the letter. The end of the letter shows oblique chatter marks, suggesting that the chisel was moved from down-up to create the final part, as seen in the experimental R. The entry points of the tool, the places where the tool stops during its movement and a change of direction are evidenced by the presence of steps, as in the case of the final section of the R. This highlights the difficulties in controlling the movements to produce the end of the letter, which are also documented in the experimental replicas (R, E (Capital) and S). Moreover, just like in the experimental letters, striations can also be observed on the archaeological letters. These striations, created by the movement of the tool, help us understand the gestures of the craftsman, especially in areas where there was a rotation of the hand, as indicated by curved striations that then become rectilinear, particularly at the ends of the letter. The differences between the two letters are also visible in the workmanship: the letter E (Gothic) from Viterbo exhibits a more refined technique with no visible errors, and the incisions seem to have been made quickly with few tool passes, indicating a skilled artisan. On the other hand, the letter R from Cencelle, though well-made, shows some defects such as partial wall collapse and deviations in the incision, likely due to quartz in the stone, although these were corrected by multiple tool passes, still demonstrating the craftsman’s skills. Both the 3D visualization and quantitative and qualitative analysis confirm the difficulty of the incision process, especially in creating the end of the letter. The artisan’s skill in both cases is undeniable. The more irregular parts and minor flaws could perhaps be attributed to the artisan’s working position and space, as well as the production time. In a workshop setting, where different commissions had to be fulfilled, time constraints were likely tight to satisfy customers as quickly as possible. In any case, any defects were not visible to the naked eye or to an untrained observer, resulting in a high-quality final product.

Finally, the 3D model and digital microscope images helped highlight any surface degradation, such as restoration materials on the letter E (Gothic) from Viterbo, which would not have been visible to the naked eye. The use of micro-photogrammetry further validated its potential as an effective tool for an initial analysis of alterations and degradation, also allowing a more precise selection of areas for the examination of traces.

## 5. Conclusions

The results obtained in identifying the tools, techniques, gestures, and work phases of the artisan demonstrate the effectiveness of the experimental and traceological approach in the analysis of epigraphic inscriptions. This highlights the importance of a methodological integration between trace analysis, experimentation, and field documentation techniques such as micro-photogrammetry. This integration helps define study and analysis protocols that can also be applied to different types of materials, while also allowing the recognition of a series of traces that made it possible to reconstruct the direction of movements, the methods of incision, and the technical skill of the artisans.

The differences observed in the execution of the archaeological letters, compared also to the experimental letters, confirm the usefulness of integrating these two types of data to interpret technical gestures and the production context, highlighting the high skill level of medieval artisans.

The potential of such an analysis is particularly evident in the epigraphic field, where the topic of production has been largely overlooked by scholars over time, introducing a significant element of novelty in research. Moreover, when extended to a broader scale that considers both historical and territorial contexts, this type of study raises further implications, questions, and perspectives that could lead to entirely new lines of research.

In the case of the epigraphs from northern Lazio, for example, a comparison with a larger set of inscriptions from the same chronological and geographical context – combined with an analysis of stone sculpture in Rome between the Early and Late Middle Ages – could provide valuable data for defining broader technological patterns. This would help to better understand the variables linked to different craft traditions, production processes, and patronage in medieval Lazio.

## Supporting information

S1 Video Phases of making the experimental letter R by the artisan. (MP4)

S2 Video Phases of making the experimental letter S by the artisan. (MP4)

S3 Video Phases of making the experimental letter C by the artisan. (MP4)

S4 Video Phases of making the experimental letter E (Capital) by the artisan. (MP4)

S5 Video Phases of making the experimental letter E (Gothic) by the artisan. (MP4)

S1 Text Experimental sheet of the making of the letter R. (DOCX)

S2 Text Experimental sheet of the making of the letter S. (DOCX)

S3 Text Experimental sheet of the making of the letter C. (DOCX)

S4 Text Experimental sheet of the making of the letter E (Capital). (DOCX)

S5 Text Experimental sheet of the making of the letter E (Gothic). (DOCX)

S1 Fig Comparison between the three methods of trace observation. At the top, view under the stereomicroscope with reflected light on the stone surface; in the center, stereomicroscope with transmitted light on the resin; and at the bottom, micro-photogrammetric view of the final part of the letter E (Capital).
(TIF)

S2 Fig Analysis of the roughness of the experimental letters C, E (Capital), and S. (TIF)

S3 Fig Slope analysis of the experimental letter C with profile extrapolation at the marked points. (TIF)

S4 Fig Slope analysis of the experimental letter E (Capital) with profile extrapolation at the marked points. (TIF)

S5 Fig Slope analysis of the experimental letter S with profile extrapolation at the marked points. (TIF)
