# Supplementary material for: From chisel to inscription: affordable protocols for the digital documentation of stone carving techniques. An experimental archaeology and traceological approach applied to epigraphy
Source: PLoS One. 2025 Jul 7;20(7):e0327303. doi: 10.1371/journal.pone.0327303 (PMC12233910; doi:10.1371/journal.pone.0327303)
Supplement: S1 Text — (DOCX) [file pone.0327303.s006.docx]

**Experimental sheet**

| N°/Letter | : | R |
| --- | --- | --- |
| Raw material | : | White marble |
| Shape of pieces | : | Square |
| Dimensions (mm) | : | 78 x 81 (letter area 30 x 39) |
| Preparation of surface before the works | : | The original surface of the slab was glossy, but before the letter carving process it was worked by the chisel and removed the polished part and became opaque. Letter sketch was made by pencil. |
| Performed activity (in brief) | : | Reproduce the Roman style letter R consists of two walls (V-shape groove) |
| Performed action (explain more detail) | : | The artisan carves the leg part of the R starts on the lower edge upwards. Minor rotation of the wrist is recognized during the construction of the small arch at the bottom of the leg and it affects chisel shifts into a wider angle (from 45° up to nearly 90°) as it arrives at the uppermost part. He works repeatedly so the leg results in two walls. At the end the artisan clean up the debris (seems like powder) with his index finger (0:00-0:44)  It is the turn for the arch shaping. It begins from the lower part (the point next to the upper part of the leg) towards the upper part of the arch. The half-round arch shaping is represented in a circular anticlockwise movement of the artisan’s (especially upper body, arm, and wrist). Chisel shifts into a wider angle detected as well and he use the extreme corner of chisel and more strikes at nearly end of the arch (upper serif) (00:46-2:16)  To form the stem, it starts with the inner wall. A slight rotation identified before the continuous and uninterrupted strikes from bottom to the upper part. He adds more strikes for the lower part of the stem using the extreme corner of the chisel (serif) (2:20-2:58). |
| The movement | : | 1. Circular move to form the arch and small arch of the leg. The circular move involves the wrist, arm, and upper body (it recognizes that he changes his position to the other side) and affects the shift of the chisel into a wider position. 2. Vertical strikes; upwards movement, for stem and leg. |
| Work duration | : | 2 minutes 58 seconds |
| Comments | : | According to the artisan, R is classified as a difficult letter.  There is no significant disruption for the piece displacement, because the artisan put a clamp on the stone. |

Tools used

| Hammer | : | Wooden hammer (the whole part; rounded head and handle) |
| --- | --- | --- |
| Chisel | : | Flat chisel |
| Part of chisel used | : | Corner of the tip (also the extreme corner), due to the small size of the letter |
| Angle of chisel | : | Maintained at 45° in general, wider angle recognized especially for shaping the arch part |
