# Supplementary material for: From chisel to inscription: affordable protocols for the digital documentation of stone carving techniques. An experimental archaeology and traceological approach applied to epigraphy
Source: PLoS One. 2025 Jul 7;20(7):e0327303. doi: 10.1371/journal.pone.0327303 (PMC12233910; doi:10.1371/journal.pone.0327303)
Supplement: S2 Text — (DOCX) [file pone.0327303.s007.docx]

**Experimental sheet**

| N°/Letter | : | S |
| --- | --- | --- |
| Raw material | : | Gray marble |
| Shape of pieces | : | Square |
| Dimensions (mm) | : | 60 x 330 |
| Preparation of surface before the works | : | No |
| Performed activity (in brief) | : | To reproduce the letter S using a chisel with a rounded end. The letter has U-shape groove. |
| Performed action (explain more detail) | : | (to simplify the explanation, we divided the S into two parts according to the arch; upper arch and lower arch)  00:03-00:43  He starts to work in the middle of the lower arch, outward beats direction identified, moves from the arch to the edge. At some moments he stops to arrange the angle at 45°. As the part has a half circular shape, his body, wrist, and arms move to follow it.  After finishing at the edge, then he begins to continue from the middle part of the lower arch upward to the upper arch. When the chisel reaches the edge of the upper arch, he rotates the stone 180° anticlockwise and resumes the rest of the upper arch from the middle of the upper arch towards the edge (outward direction). So, now the form of the S is complete.  00:45 - 01:29  Works on the upper edge with several stable strikes and continuously refines the upper arch (inwards motion, from the edge to the arch). Again, he rotates the piece 180° clockwise then gives more strikes from the end of the upper arch, towards the diagonal line of the S and the middle part of the lower arch. Another 180° anticlockwise rotation attempts, then he begins to clarify the engraving from the middle of the upper arch until its edge. Then he blows the dust out of the surface.  01:30 - 01:45  From the middle part of the upper arch, he strikes towards the diagonal line to the middle part of the lower arch. An interruption recognized as the stone piece moves and displaced. Then he needs to relocate the position and rotate it 90° anticlockwise (at this orientation the lower arch facing up and the upper arch facing down/towards the artisan).  01:46 - 02:17  Continuous strikes applied from the middle of the lower arch into its edge, followed by several strikes focusing on the edge using the corner and the rounded part of the chisel. He takes 180° anticlockwise rotation and does similar strikes for the edge of the upper arch before rotating the piece again in 90° anticlockwise.  02:18 - 02:56  The current position represents inverted S where the lower arch is at the top. The artisan refines the upper arch towards the edge several times. He also clarified the diagonal part with the upward motion, then again clarified the upper arch into the edge and rotated the stone 90° clockwise.  02:57 - 03:30  The upper arch is now facing up. He put emphasis on strikes forming the edge of the upper arch. Then, he gives more strikes for the lower arch towards the diagonal part and continues into the upper arch directly without interruption. After that, he rotates the piece 90° anticlockwise back into the initial position where he begins the S formation. He adds more strikes from the end of the diagonal part towards the lower arch to the end. |
| The movement | : | Variation of movement applied due to the shape of the letter including circular and diagonal. The motion can be inwards or outwards depending on the piece orientation. |
| Work duration | : | 3 minutes 30 seconds |
| Comments | : | Rounded chisel used is longer than the flat chisel. Rotation attempted many times in 90° and 180°. The piece is not retained by the clamp, but another stone piece caused a displacement. |

Tools used

| Hammer | : | Squared metal head hammer with wooden handle |
| --- | --- | --- |
| Chisel | : | Rounded tip chisel |
| Part of chisel used | : | The whole tip, the corner of the tip for constructing the edge |
| Angle of chisel | : | Maintained at 45° |
