# Supplementary material for: From chisel to inscription: affordable protocols for the digital documentation of stone carving techniques. An experimental archaeology and traceological approach applied to epigraphy
Source: PLoS One. 2025 Jul 7;20(7):e0327303. doi: 10.1371/journal.pone.0327303 (PMC12233910; doi:10.1371/journal.pone.0327303)
Supplement: S3 Text — (DOCX) [file pone.0327303.s008.docx]

**Experimental sheet**

| N°/Letter | : | C |
| --- | --- | --- |
| Raw material | : | Tuff (Peperino), unsorted grains, dark gray color, unpolished surface |
| Shape of pieces | : | Square |
| Dimensions (mm) | : | 107 x 111 |
| Preparation of surface before the works | : | No, only letter sketch using pencil on the surface |
| Performed activity (in brief) | : | Constructing the C with inner and outer walls to have a V-shape groove using flat surface of chisel |
| Performed action (explain more detail) | : | Carving process starts from the lower edge upward to the middle part of the arch for the inner wall. It stops for a while, then begins again from the middle to the upper part (0:00 - 0:27).  Before reaching the exact upper edge, the artisan cleans the debris and rotates the stone 90° anticlockwise and finishes the rest right after. The utilization of the chisel's extreme corner is recognized to shape the inner wall of the upper edge part (0:30 - 0:44).  Then, he works on outer walls, from the middle part of the arch to the upper edge. He also refined the edge of the upper edge with a slight circular motion (0:49 - 1:12).  Several strikes are performed to carve the outer wall from the middle part of the arch to the lower edge (1:13 - 1:22). Another rotation in 90° anticlockwise is detected and he extends a deeper edge using the extreme corner of the chisel for the lower edge. Other strikes stressed the middle part of the arch and continuously reached the rest of the lower part, then removed the debris (1:24 - 1:54).  He rotates the stone 180° anticlockwise, so it remains as the initial position. The strikes concentrated on the middle part of the arch for both walls. Then, before work ends, he works on the outer wall for the middle arch to the upper edge (1:56 - 2:25). |
| The movement | : | 1. Circular strikes follow the shape of the C. Working direction is diverse due to the stone rotation. 2. Chisel shifting; from the flat to the extreme corner for the edge shaping. |
| Work duration | : | 2 minutes 25 seconds |
| Comments | : | The artisan needed to clean the debris, and it caused strikes interruption. The stone sometimes moves because it is only retained with another stone piece. |

Tools used

| Hammer | : | Squared metal head hammer with wooden handle |
| --- | --- | --- |
| Chisel | : | Flat chisel |
| Part of chisel used | : | Mostly half or the whole flat part, sometimes the extreme corner |
| Angle of chisel | : | Maintained at 45° |
