# Supplementary material for: From chisel to inscription: affordable protocols for the digital documentation of stone carving techniques. An experimental archaeology and traceological approach applied to epigraphy
Source: PLoS One. 2025 Jul 7;20(7):e0327303. doi: 10.1371/journal.pone.0327303 (PMC12233910; doi:10.1371/journal.pone.0327303)
Supplement: S4 Text — (DOCX) [file pone.0327303.s009.docx]

**Experimental sheet**

| N°/Letter | : | E (Capital) |
| --- | --- | --- |
| Raw material | : | White marble |
| Shape of pieces | : | Square |
| Dimensions (mm) | : | 118 x 113 |
| Preparation of surface before the works | : | No, only letter sketch using pencil on the surface |
| Performed activity (in brief) | : | Reproduce the letter E (Capital). The expected result is a letter with square groove. |
| Performed action (explain more detail) | : | Using a wooden hammer and flat chisel, the artisan begins to form the lower edge of the E. It is evaluated that forceful and continuous strikes applied due to the hardness of marble. He works from right to left (inwards) and also in reverse. He gives some details for the border using the corner of the chisel. The chisel side exchanged efficiently by moving his little finger (00:00 - 00:46)  To form the stem direction of strikes is from bottom to top. This step covers the previous trace at the intersection between lower arm and stem. At some moments he changes the wooden hammer into the metal head (rounded) hammer and repeats the strikes for the stem (00:52 - 01:07)  Middle arm also worked by inward strikes. After a series of strikes, he again gives more inward strikes to the lower arm and passes the lower part of the stem. Then other strikes attempt for the stem, upward with a slight pause in the middle part (01:12 - 01:48)  Vertical strikes (upward and downward) recognized on the edge of the middle arm’s width. It follows by horizontal inward strikes on the arm’s length and repeat (01:50 - 02:13)  Again, he works on the lower arm (inward) and stem (upward) (02:14 - 02:21),  The work continues on the upper arm for the first time applying inward strikes. Upward and downward strikes are recognized for the upper arm’s width. Inward strikes repeat a few times and sometimes start from the middle of the stem. This last part of work gives an emphasis for the upper arm, however he also works at the stem of the upper arm area and from the middle upward (02:23 - 03:17) |
| The movement | : | Horizontal, vertical, reverse direction (for example, right to left then left to the lower part of stem, upward to downward then downward to upward for the middle arm) |
| Work duration | : | 3 minutes 8 seconds |
| Comments | : | The hardness of marble causes the artisan to intend to change the hammer, no interruption since the slab retained using metal clamp |

Tools used

| Hammer | : | 1. Wooden hammer (the whole part; rounded head and handle) 2. Rounded metal head hammer with wooden handle |
| --- | --- | --- |
| Chisel | : | Flat chisel |
| Part of chisel used | : | Whole tip/ flat part (for most of the time)  Extreme corner/incisive part (to refine the border) |
| Angle of chisel | : | 60° (in general)  45° to 90° (to refine the border) |
