# Supplementary material for: From chisel to inscription: affordable protocols for the digital documentation of stone carving techniques. An experimental archaeology and traceological approach applied to epigraphy
Source: PLoS One. 2025 Jul 7;20(7):e0327303. doi: 10.1371/journal.pone.0327303 (PMC12233910; doi:10.1371/journal.pone.0327303)
Supplement: S5 Text — (DOCX) [file pone.0327303.s010.docx]

**Experimental sheet**

| N°/Letter | : | E (Gothic) |
| --- | --- | --- |
| Raw material | : | White marble |
| Shape of pieces | : | Square |
| Dimensions (mm) | : | 29 x 15 |
| Preparation of surface before the works | : | No |
| Performed activity (in brief) | : | Reproduce gothic style E. It has a distinction from the medieval or Roman E letter where the arch substitutes for the vertical stem and the three arms are closed by a vertical line. The appearance is more identical to the D letter in a mirror view. However, it has the v-shape groove as the Roman style letter. |
| Performed action (explain more detail) | : | The artisan constructs the arch (which remains as the stem in another E/ medieval or Roman). The starting point is not exactly at the edge, but at a point between the edge of the vertical line and before the curve. The motion is upward, from the lower to the upper part. It also ends before reaching the edge, repeated; once to shape the inner wall and once for the outer wall. The constant circular motion for the arm and wrist is discernible. On this state the letter is identical to the mirror view of D. He cleans the dust and rotates the slab 90° anticlockwise (00:00 - 00:31)  Now the arch stands at the bottom and the vertical line facing up. He works from the middle of the arch upward to the right (toward the lower part); this motion is applied to develop the inner and outer wall. Similar action repeated for the other side and stressing for the edge of both arms using the corner of the chisel (00:34 - 01:27)  The middle arm is made from the middle of the arch upward to the middle of the line. He commenced by creating a diagonal point using the corner of the chisel to have an impression of depth. Then he continues to engrave the middle arm with two walls (repetition and dust cleaning recognized, follows by 90° clockwise rotation, so the slab is now back at the initial position) (01:28 - 01:50)  After it remains as the original position, the artisan checks if the result is already complete. He decides to refine the arch’s outer wall by a constant upward strike, then rotate the piece again in 90° anticlockwise (01:51 - 02:09)  He gives more strikes for the inner wall and the edge (of the upper arm), a wider angle of chisel detected as it reaches the edge. Another 90° anticlockwise rotation attempts, so now the letter looks like D (02:11 - 02:17)  At this position he clarified the vertical line. Starting at the edge (which belongs to the upper arm), he utilizes the extreme corner of the chisel then follows with upward strikes towards the other arm. The angle shifting is noticeable. First, the edge (of upper arm) uses wider angle, then smaller for the line, and extended wider as he arrived at the other side of the edge (of lower arm) (02:21 - 02:36) |
| The movement | : | 1. Vertical, horizontal. 2. Circular motion is evaluated from the artisan’s wrist and arm. |
| Work duration | : | 2 minutes and 36 seconds (approximately) |
| Comments | : | Video recorded after the vertical line and the arch are done. The slab is retained by a bigger marble fragment, so the interruption of displacement is almost absent. |

Tools used

| Hammer | : | Rounded metal head hammer with wooden handle |
| --- | --- | --- |
| Chisel | : | Flat chisel |
| Part of chisel used | : | Corner |
| Angle of chisel | : | Maintained at 45°, shift into a wider angle detected for the edges shaping |
