# Supplementary figures and images for: From chisel to inscription: affordable protocols for the digital documentation of stone carving techniques. An experimental archaeology and traceological approach applied to epigraphy
Source: PLoS One. 2025 Jul 7;20(7):e0327303. doi: 10.1371/journal.pone.0327303 (PMC12233910; doi:10.1371/journal.pone.0327303)

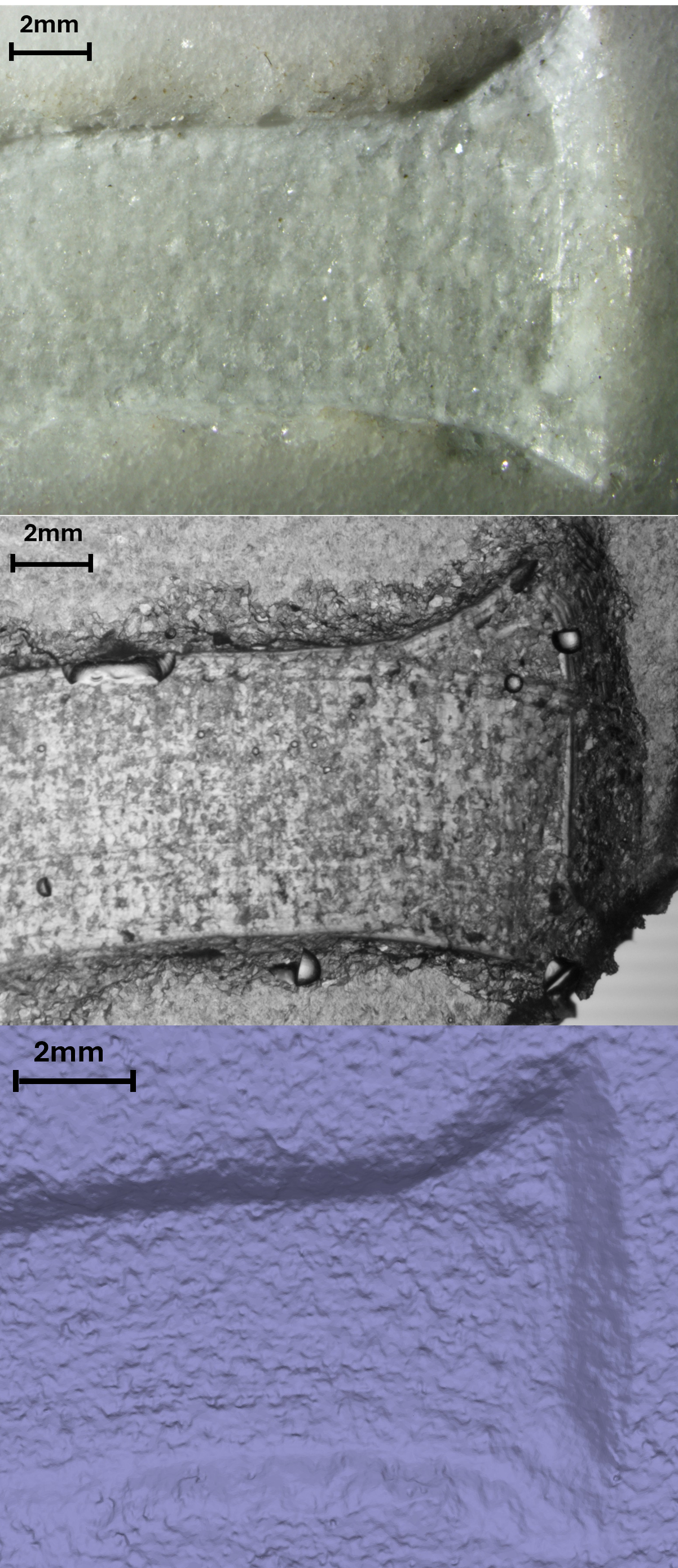

Supplement: S1 Fig — At the top, view under the stereomicroscope with reflected light on the stone surface; in the center, stereomicroscope with transmitted light on the resin; and at the bottom, micro-photogrammetric view of the final part of the letter E (Capital). (TIF) [file pone.0327303.s011.tif]

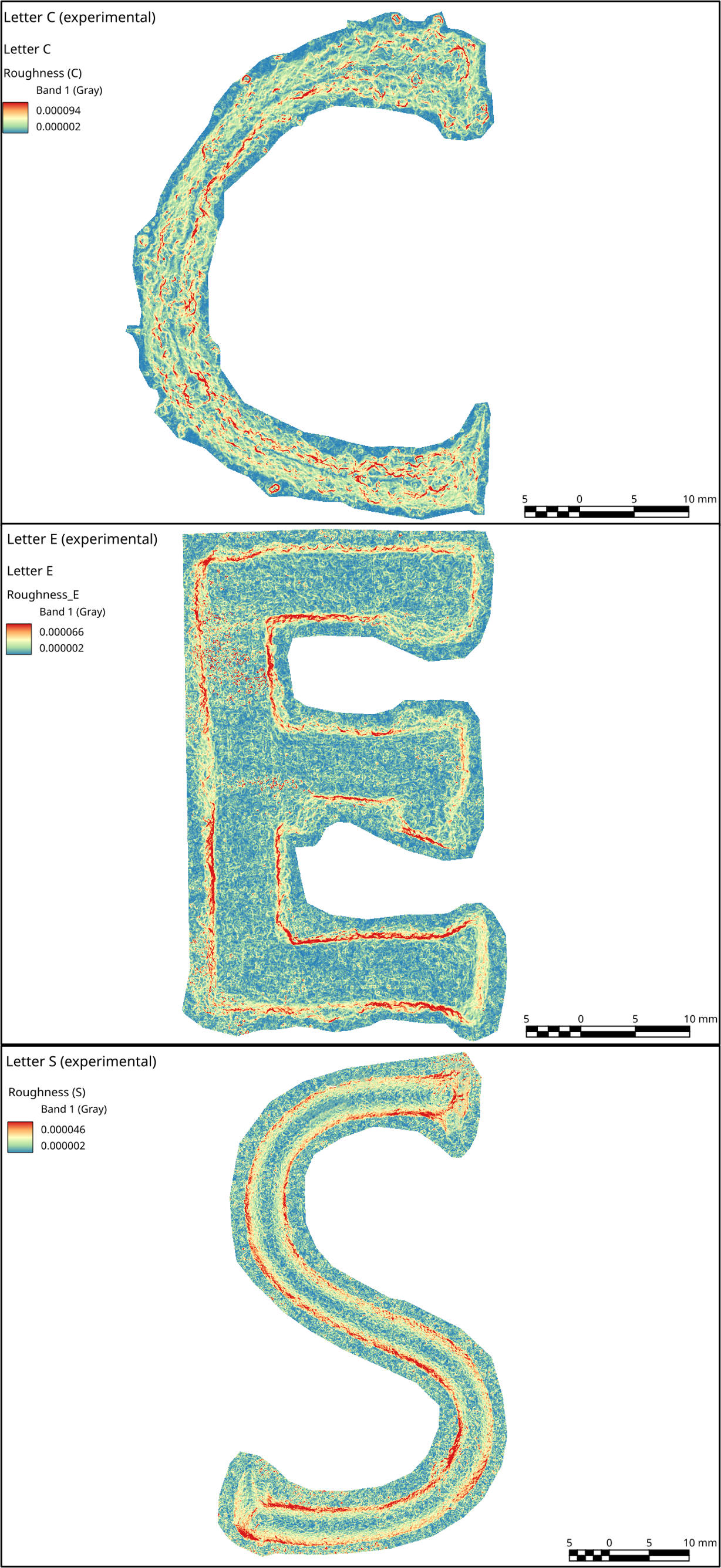

Supplement: S2 Fig — (TIF) [file pone.0327303.s012.tif]

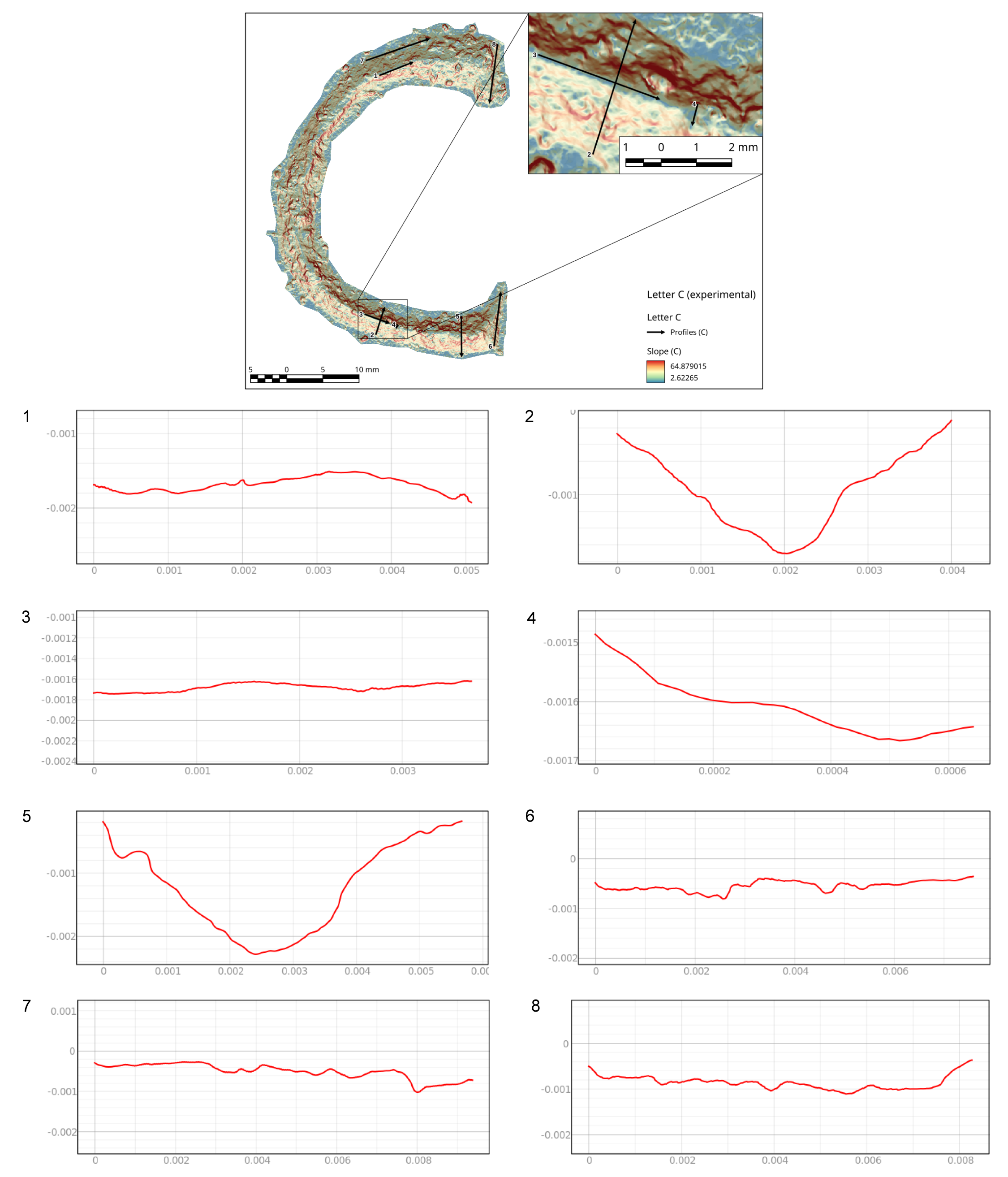

Supplement: S3 Fig — (TIF) [file pone.0327303.s013.tif]

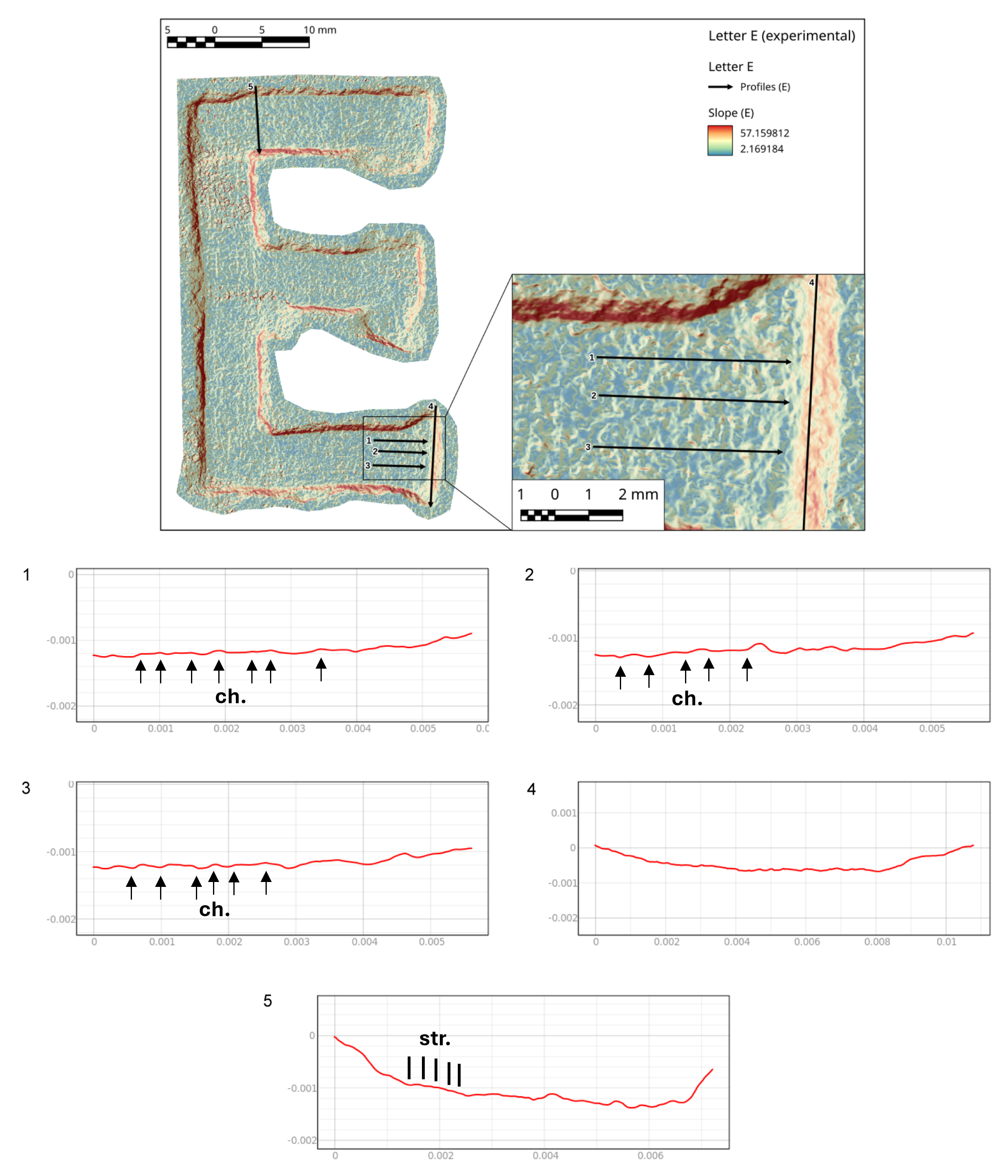

Supplement: S4 Fig — (TIF) [file pone.0327303.s014.tif]

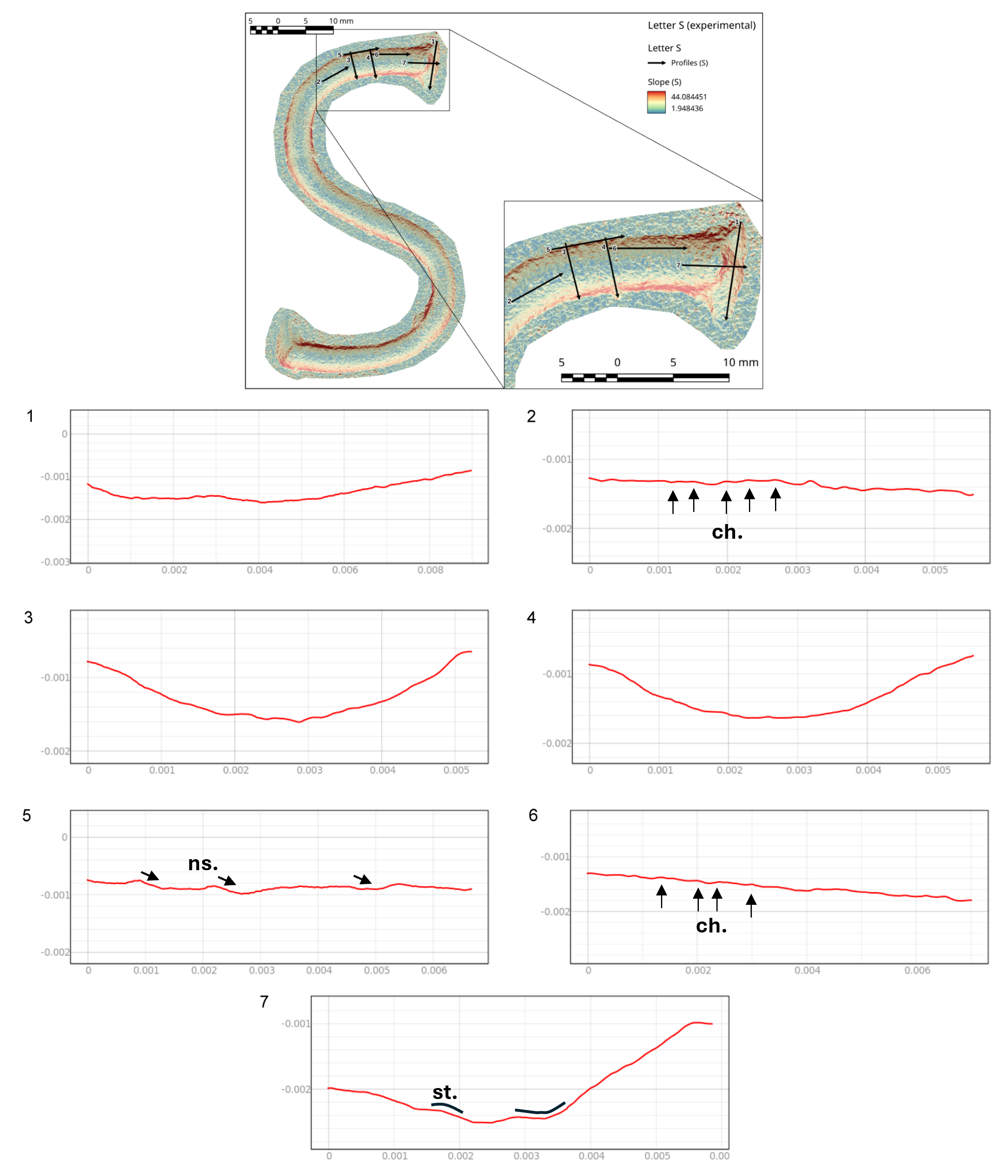

Supplement: S5 Fig — (TIF) [file pone.0327303.s015.tif]
